# Validation of an instrument for patient classification to support obstetric nursing care

**DOI:** 10.1590/0034-7167-2023-0401

**Published:** 2024-07-19

**Authors:** Vanessa Damasceno Laporte, Clara Fróes de Oliveira Sanfelice, Ariane Polidoro Dini

**Affiliations:** I Universidade Estadual de Campinas. Campinas, São Paulo, Brazil

**Keywords:** Validation Study, Personnel Management, Nursing Assessment, Obstetric Nursing, Hospital Administration, Estudio de Validación, Administración de Personal, Evaluación en Enfermería, Enfermería Obstétrica, Administración Hospitalaria

## Abstract

**Objectives:**

to develop and validate an instrument for the classification of pregnant and postpartum women according to the demand for nursing care.

**Methods:**

a methodological study conducted in three stages: 1) construction of the instrument based on literature review; 2) content validation using the Delphi technique with 12 experts; and 3) Evaluation of the convergent construct validity through the correlation between the scores of the constructed instrument and the Fugulin instrument.

**Results:**

an instrument with ten indicators of specific care for pregnant and postpartum women was developed. A content validity index higher than 0.9 was obtained, requiring only one round of the Delphi technique. The Spearman coefficient was 0.64 between the instruments, indicating a strong correlation.

**Conclusions:**

the classification instrument specifically constructed for pregnant and postpartum women showed evidence of content validity and convergent construct validity with a widely used instrument in the national territory.

## RESUMEN

**Objetivos:**

construir y validar un instrumento para clasificación de gestantes y puérperas segundo la demanda de cuidado de enfermería.

**Métodos:**

estudio metodológico realizado en tres etapas: 1) construcción del instrumento basado en la revisión bibliográfica; 2) validación de contenido aplicando la técnica Delphi con 12 especialistas; y 3) Evaluación de la validez de constructo convergente mediante la correlación entre los escores del instrumento construido y del instrumento de Fugulin.

**Resultados:**

fue construido un instrumento con diez indicadores de cuidados específicos para gestantes y puérperas. Obtenido el índice de validez de contenido superior a 0,9, siendo necesaria sólo una rodada de la técnica Delphi. El coeficiente de Spearman fue de 0,64 entre los instrumentos, indicando una correlación fuerte.

**Conclusiones:**

el instrumento de clasificación construido específicamente para gestantes y puérperas presentó evidencias de validez de contenido y validez de constructo convergente con instrumento ampliamente utilizado en territorio nacional.

## INTRODUCTION

The gravid-puerperal cycle brings significant changes to the personal and family life of women. In addition to the physical and psychological alterations inherent to this period, there are changes in the family and social environment^(1)^.

Despite being a physiological cycle, certain situations make it more susceptible to unfavorable outcomes, such as complications arising from the gestational process^(2)^. According to the Ministry of Health (MS)^(3)^, approximately 10% of pregnancies in Brazil are considered high risk. A pregnancy may be deemed high risk due to various factors, including: arterial hypertension, diabetes, hemorrhagic syndromes, infectious diseases, advanced age or teenage pregnancy, neoplasms, a history of previous preterm birth or fetal malformation, and individual and sociodemographic characteristics that pose a risk to the pregnant woman and fetus, among any other condition that endangers maternal and/or fetal health. In some cases, women need to be admitted to obstetric beds for monitoring or clinical treatment. Studies indicate that women requiring hospitalization during pregnancy for obstetric complications are more likely to experience negative maternal and perinatal outcomes^(2)^.

The care provided to high-risk pregnant women during hospitalization has a significant impact on women’s survival. The lack of planning for the provision of such care can delay the implementation of necessary measures to reduce the risk of maternal death and improve the quality of care for pregnant women^(4-7)^.

Aiming for the efficient performance of the care team, encompassing the identification of problems that may result in greater harm to the health of women and their children, the use of discriminating instruments in the process of recommending, generating, and providing differentiated care is necessary^(5)^.

Due to their active participation in the care of this audience, nursing plays a prominent role in this function. In Brazil, the Federal Nursing Council (Cofen) regulates the calculation for the sizing of nursing professionals, considering patient classification^(8)^. Using validated instruments for patient classification is recommended so that the specificities of those receiving nursing care are considered^(9-10)^.

Given the absence of patient classification instruments specific for pregnant and postpartum women hospitalized in obstetric inpatient units, the Fugulin instrument^(11)^ has commonly been used for this purpose. Generally, this instrument does not allow for the assessment of the specificities of care for pregnancy and the puerperium. Even though there is a tool that classifies the mother and the rooming-in baby according to the demand for nursing care, the individual classification of the woman in her gravid-puerperal period cannot be performed by the binomial assessment instrument^(12)^.

Thus, the construction and validation of an instrument that allows the classification of pregnant and postpartum women according to the demand for nursing care aims to fill a gap in the literature, contributing directly to the qualification of obstetric nursing care, making it more appropriate and safe. Moreover, it helps to reduce maternal mortality, which is one of the Millennium Development Goals^(13)^, and aligns with the priorities of the National Agenda for Health Research Priorities^(14)^, where the gravid-puerperal cycle is one axis of study.

## OBJECTIVES

To construct and validate an instrument for classifying patients according to the demand for nursing care for pregnant and postpartum women.

## METHODS

### Ethical Aspects

The study adhered to the recommendations of Resolution 466/12 and was approved by the Ethics and Research Committee of the hospital where the research was conducted. All experts participating in the content validation phase signed the Informed Consent Form before participating in the study.

### Study Design, Period, and Location

It is a methodological study conducted in a tertiary complexity hospital in São Paulo, Brazil, renowned for women and newborn healthcare. The study took place in three phases, from October 2020 to August 2022:

1. Construction of the instrument (from September to December 2021);
2. Evaluation of content validity evidence through the Delphi technique (from January to February 2022);
3. Evaluation of convergent construct validity by correlating the scores of the constructed Pregnant and Postpartum Women Classification Instrument with the Fugulin instrument^(11)^, in a sample from an obstetric inpatient unit (from April 2022 to August 2022).

### Inclusion and Exclusion Criteria

In the stage of evaluating content evidence through the Delphi technique, the inclusion criteria for selecting experts were: nurses experienced in instrument validation, with published articles in the area, or experience in nursing care or management of obstetric inpatient units for two years or longer. Exclusion criteria were: lack of interest or understanding to judge the instrument’s content; disagreement with signing the Informed Consent Form of the approved research protocol; and less than two years of experience in the care and management of obstetric inpatient units.

The study’s researcher, who has been providing care at the collection site for nine years, conducted the data collection procedure for the instrument’s pre-test. Each obstetric inpatient bed was classified only once with each instrument on the same day and time of the application. At this stage, the following inclusion criterion was considered: beds occupied by pregnant and postpartum women who were not in rooming-in accommodation.

### Study Protocol

The process for constructing the Pregnant and Postpartum Women Classification Instrument (*Instrumento de Classificação de Gestantes e Puérperas* - ICGP) was based on: obstetric nursing references^(1-7)^ to identify potential areas generating demand for nursing care in pregnant or postpartum women; previously validated patient classification instruments^(9-12,15-17)^ to support the assessment structure of factors, i.e., indicators of nursing care needs, objectively analyzed by a four-point ordinal scale, organized in ascending order according to care demand; and legislation for Nursing Professionals Sizing^(8)^, which underpinned the number of care categories in the instrument and the number of nursing hours per day for each care category.

Following the instrument’s construction, its validation process began using the Delphi technique^(17-18)^, where expert nurses meeting the established inclusion criteria assessed the instrument through an electronic questionnaire. The set content validity index (CVI) was 0.8, with data collection rounds repeating as necessary until this ideal CVI was reached^(17-18)^.

The questionnaire consisted of a question to evaluate the instrument’s dimensionality, agreement, and clarity; a question related to the level of care demand for each indicator; and an open space for suggestions. For validity evidence evaluation, through the CVI, experts assigned to each instrument indicator one of four options: “1) Not relevant or lacks clarity for assessing nursing care demand; or 2) Needs major revision to be relevant or clear in assessing nursing care demand; or 3) Needs minor revision to be relevant or clear in assessing nursing care demand; or 4) Relevant and representative in assessing nursing care demand”.

After consensus was reached on the number of ICGP indicators, the total score obtained from adding the maximum score of all indicators and dividing the range by 5 was considered, according to the number of care categories regulated by Cofen in the Resolution on nursing professionals sizing: minimal care, intermediate care, high-dependency care, semi-intensive care, and intensive care.

Subsequently, the final version of the ICGP was applied to 120 beds in the obstetric inpatient unit of a university hospital in the state of São Paulo. The same evaluator, who has experience in the unit and is one of the study’s authors, carried out the ICGP application, mainly due to limited access to the data collection site during the pandemic period. It is important to note that the data collection spreadsheet considered the application of both the ICGP and the Fugulin instrument^(11)^, already applied daily at the study site. Only beds occupied by pregnant or postpartum women not in rooming in accommodation were taken into account.

### Analysis of Results and Statistics

All responses obtained in the first phase of the Delphi technique were extracted from the response platform and tabulated in an electronic spreadsheet to calculate the CVI, according to the formula^(19)^: CVI = ∑ Number of responses “3” or “4”/ Total number of responses.

The data related to the application of the ICGP on the 120 beds were tabulated in an electronic spreadsheet, and the database was analyzed using the statistical software SPSS version 23, to perform the calculation of the instrument’s convergent construct validity.

Construct validity represents the degree to which the scores of an instrument are consistent with hypotheses based on the assumption that an instrument validly measures the construct it is intended to measure^(20)^.

For this assessment, the Spearman correlation coefficient^(21)^ was analyzed, a non-parametric measure of the statistical correlation between the scores of two variables - in this case, the ICGP and the Fugulin instrument^(11)^. Values greater than or equal to 0.50 are indicative of a strong correlation between the analyzed variables.

## RESULTS

After reviewing the literature, a “Version 0” of the ICGP consisting of ten care indicators was developed: Mental state, behavior, and/or mood; Respiratory support; Interval for vital sign measurement; Dependence for daily life activities; Nutritional and hydration support; Drug therapy; Eliminations; Skin-mucous membrane integrity; Breast care; Family support network and understanding of health status. All indicators were composed of a 1 to 4 point Likert-type scale about the level of nursing care demand, with the lowest demand being 1 and the highest corresponding to 4 points.

Of the 15 specialist nurses invited to judge the content of the instrument, only 12 agreed to participate in the first phase of the study. The characteristics of the participants are presented in Table 1. The response deadline was set at 15 days, but each questionnaire was answered within three days.

**Table 1:** Characterization of Experts Participating in the Evaluation of the Pregnant and Postpartum Women Classification Instrument (N = 12), Campinas, São Paulo, Brazil, 2023

Characterization	n	%
Age		
28-30	1	8%
31-40	4	33%
>40	7	58%
Years of Training		
6-10	4	33%
11-19	3	25%
20-27	5	42%
Professional Experience (years)		
4-8	2	17%
9-19	5	42%
20-26	5	42%
Field of Work		
Direct Care	8	67%
Management	3	25%
Teaching and Research	1	8%
Professional Qualification		
Undergraduate Degree Only	1	8%
Specialization	10	83%
Master's Degree	4	33%
Doctorate Degree	3	25%

The data obtained from the experts’ responses in this first phase of the Delphi technique are presented in Table 2.

**Table 2:** Percentage of Agreement on Indicators and Graded Situations of the Pregnant and Postpartum Women Classification Instrument (N = 12), Campinas, São Paulo, Brazil, 2023

Indicator	Care Indicators	Graded Situations
Family Support Network	100%	100%
Mental State, Behavior, and/or Mood	100%	91.7%
Dependence for Daily Life Activities	100%	100%
Breast Care	100%	100%
Skin-Mucous Membrane Integrity	100%	100%
Nutritional and Hydration Support	100%	100%
Eliminations	100%	100%
Respiratory Support	100%	100%
Interval for Monitoring Controls	100%	100%
Drug Therapy	100%	100%

All judges marked options 3 or 4 for content analysis; thus, the CVI for all indicators was equal to 1. Besides these objective responses, some experts provided suggestions for better understanding the instrument, which were analyzed, and the researchers accepted those deemed relevant.

For the “Mental state, behavior, and/or mood” indicator, one expert suggested replacing the term “multicomplaintive” with “multiple complaints”. In addition, for this indicator, another researcher suggested that the option graded as number 2 be adapted from “Oriented and/or slightly anxious or complaining” to “Oriented, yet anxious or complaining, and/or social withdrawal” since the patient’s spatial orientation and behavior are related but independent assessments. The text was rewritten in its final version as: “2 - Oriented, anxious or complaining; and/or with social withdrawal”.

In the “Respiratory support” indicator, two judges pointed out the need to replace the word “tracheostomized” with “with tracheostomy” in the fourth grade: “4 - In need of invasive or non invasive mechanical ventilation; and/ tracheostomized”. The suggestion was accepted, and the final version became: “4 - In need of invasive or non-invasive mechanical ventilation; and/ with tracheostomy”.

To revise the “Dependence for daily life activities” indicator, in grades 1 and 2, the suggestion of two experts to include observation of the need for orientation and assistance was accepted.

The changes made to the “Eliminations” indicator related to the spelling of the words “WC” in grade number 2 and “Ostomies” in grade 4, following conceptual updates. The suggestions were accepted, and the rewriting was as follows: “2 - Requires assistance for bathroom eliminations and/or altered frequency of urinary and intestinal eliminations”; and “4 - Use of diaper, urinary catheter, and stomas”.

Given the agreement of over 90% on all indicators and graded situations, as well as a CVI greater than 0.9, there was no need for another round of the Delphi technique. Based on this result, the final version of the ICGP was constituted of ten nursing demand indicators, with their respective conceptual definitions. For each indicator, four graded situations from 1 to 4 points were established, increasing according to the nursing demand (Chart 1).

**Chart 1:** Pregnant and Postpartum Women Classification Instrument, Campinas, São Paulo, Brazil, 2023

Family Support Network and Understanding of Health Status: Need for guidance and support for the pregnant woman, postpartum mother, or family member involved in care. 1 - Pregnant or postpartum woman has family support; understands and follows the health team’s guidance for care during hospitalization or in preparation for discharge. 2 - Pregnant or postpartum woman with or without family support; and/or has difficulties in understanding and/or following the health team’s guidance for care during hospitalization or in preparation for discharge. 3 - Pregnant or postpartum woman has difficulties adhering to guidance and/or with a social or family support network that hinders following the health team’s guidance during hospitalization or in preparation for discharge. 4 - Pregnant or postpartum woman with an absent social or family support network or one that prevents adherence to the health team’s guidance during hospitalization or in preparation for discharge.
**Mental State, Behavior, and/or Mood:** Assessment of orientation conditions, non-verbal communication of the activity level, and/or emotional state of the pregnant or postpartum woman. 1 - Oriented in time, space, and person; and calm. 2 - Oriented, yet anxious or complaining; and/or with social withdrawal. 3 - Confused or drowsy; and/or in an anxiety crisis; or with multiple complaints; and/or with excessive irritability. 4 - Unconscious and/or sedated; shows despair; and/or destructive behavior; and/or grief.
**Dependence for Daily Life Activities:** Need for help/care to move, walk, cleanse, dress. 1 - Independent in daily life activities, walks, bathes, and performs oral hygiene without needing guidance or assistance. 2 - Independent in daily life activities, walks, bathes, and performs hygiene, however, requires guidance and assistance for carrying out these activities. 3 - Requires relative rest and/or depends on a wheelchair and/or orthoses for safe mobility and needs professional help to bathe and perform oral hygiene. 4 - Completely dependent on nursing for daily life activities due to being bedridden/contained/in absolute rest; needs to be bathed and have oral hygiene performed in bed.
**Breast Care:** Need for breast care in preparation for breastfeeding, lactation suppression, and/or complications related to infection or pathologies. 1 - Breasts flaccid without hyperemia or with effective lactation inhibition when indicated. 2 - Breasts full with colostrum/milk upon expression without turgidity or pain. 3 - Breasts with signs of mammary engorgement, without signs of infection, needing milk expression performed by the postpartum woman. 4 - Breasts with signs of infection or hyperalgesic reaction or mammary fissure, needing frequent relief milking care by nursing.
**Skin-Mucous Membrane Integrity:** Need for care to maintain or repair the integrity of the skin or mucous membranes. 1 - Skin and mucous membranes intact across the entire body surface, without needing nursing care for repair. 2 - Skin dehydrated or with hyperemia or clean surgical wound without inflammatory signs, needing simple and specific care for skin lubrication or small dressing. 3 - Surgical wound and/or lesion needing dressing with repeated care renewal throughout the day and/or skin and mucous membrane care time exceeding 15 minutes. 4 - Surgical wound and/or lesion needing complex dressing with skin and mucous membrane care time exceeding 30 minutes.
**Nutritional and Hydration Support:** Need for assistance/care to ingest food and/or liquids and/or require enteral or parenteral support to meet daily needs. 1 - Self-sufficient in oral intake of liquids and foods, without assistance. 2 - Oral intake of liquids and foods with assistance. 3 - Intake of liquids and nutrients through tubes; and/or nausea/vomiting. 4 - Need for parenteral nutrition and hydration.
**Eliminations:** Need for assistance/care to perform eliminations physiologically or through devices. 1 - Self-sufficient, normal urinary and bowel elimination frequency, without needing assistance. 2 - Needs assistance for eliminations in the bathroom and/or altered urinary and bowel elimination frequency. 3 - Use of bedpan for eliminations and/or performing fluid balance and/or urinary output control. 4 - Use of diaper, urinary catheter, and stomas.
**Respiratory Support:** Need for the pregnant or postpartum woman to require assistance for airway clearance and/or oxygen supplementation. 1 - In-room air, without needing assistance for airway clearance. 2 - In-room air, with need for assistance/guidance for airway clearance. 3 - With need for oxygen supplementation, via catheter or mask. 4 - With need for invasive or non-invasive mechanical ventilation; and/or with tracheostomy.
**Interval for Monitoring Controls:** Need for observation and control of data such as vital signs, oxygen saturation, capillary blood glucose, edema, fluid balance, fetal movement assessment, vaginal losses, uterine height, or dynamics. 1 - Controls at intervals equal to or greater than every 6 hours. 2 - Controls at average intervals of every 4 hours. 3 - Controls at average intervals of every 2 hours. 4 - Continuous monitoring and/or controls at intervals less than every 2 hours.
**Drug Therapy:** Need to receive drugs for treatment or control of symptoms/underlying pathologies. 1 - Medications orally. 2 - Medications via subcutaneous, intramuscular, and/or intermittent intravenous route. 3 - Medications via continuous intravenous route. 4 - Need for vasoactive drugs and/or sedation and/or blood products.
**Classification of the Pregnant or Postpartum Woman** After evaluating the woman on all care indicators, compare with the classification scale to identify the care category: Between 10 and 15 points: minimal care Between 16 and 21 points: intermediate care Between 22 and 27 points: high-dependency care Between 28 and 33 points: semi-intensive care More than 40 points: intensive care

Of the beds, 90% were classified by the ICGP as patients requiring minimal nursing care. According to the Fugulin instrument, 97.5% of the total evaluated beds represented this category of nursing care. Both instruments classified the remaining percentage of beds as patients requiring intermediate care: 10% of the total by the ICGP and 2.5% by the Fugulin instrument. No bed was classified into the other care categories in the evaluated sample.

Regarding the analysis of the ICGP’s convergent construct validity, through the comparison between the Fugulin and ICGP instruments, a Spearman coefficient of 0.64 was obtained. This indicates a positive and strong relationship between the scores of the instruments, that is, in both instruments, the higher the score obtained, the greater the degree of complexity related to the demand for nursing care. This result demonstrates the convergent construct validity of the ICGP.

## DISCUSSION

The development of the instrument for classifying pregnant and postpartum women was based on the structure of previously validated patient classification instruments and current legislation for the sizing of nursing staff^(8,11-12,16)^. It was also driven by the need to create a specific instrument for pregnant and postpartum patients that adequately classifies this group, considering pregnancy and the postpartum period as a process in women’s health requiring specific attention^(3-4,13-14)^.

The instrument’s construction was based on the Federal Nursing Council’s legislation, which establishes the minimum amount of nursing care hours per day across five care categories: 4 hours for minimal care, 6 hours for intermediate care, 10 hours for high-dependency or semi-intensive care, and 18 hours for intensive care^(8)^.

The use of the instrument, as indicated by the legislation^(8)^, can be carried out daily as part of the nurse’s work process to assess the care demand of each ward, allowing for more assertive planning of care, favoring the balance between care supply and demand. It also serves as a basis for justifying daily reallocations, as well as allowing for the analysis of historical series and the calculation of staffing sizing.

For validation, the Delphi technique was chosen for its characteristic of preserving the confidentiality of participants’ identities and the possibility of written contributions by experts with divergent and complementary knowledge for the instrument’s construction. This technique allows consensus to be derived from reflection on the proposed issues beyond evaluating clarity and relevance^(19-20,22-25)^.

Although the literature presents the Delphi Technique’s disadvantage of the lengthy time between sending and returning questionnaires and the statistic that only 50% of those invited respond to the first contact^(19-20,22-25)^, this study achieved an 82% return rate from invitees in less than 15 days, and all responded to all proposed questions.

Regarding the selection of experts (essential for achieving higher quality in the Delphi technique execution)^(19-20,22-25)^, the heterogeneity of experience and training in the expert group composition was crucial. It allowed the instrument to be evaluated from the perspectives of care, management, and nursing research in obstetric units.

It is emphasized that heterogeneous expert groups tend to produce more qualified and acceptable solutions. Furthermore, given the divergence in the literature regarding the judges’ expertise level^(19-20,22-25)^, establishing the inclusion criterion that experts have at least two years of experience in care, management, and/or research in the obstetric field was essential. This criterion was justified by the fact that the nurses’ experience allows for a more careful examination of the ICGP.

The number of experts in Delphi technique studies can vary, but research indicates that ten or more experts, as used in this study, are optimal for consensus and relevance analysis^(19-20,22-25)^.

Using online questionnaires for data collection enabled gathering opinions from professionals across different locations and experiences, offering a broad exposition of experiences according to the reality of obstetric units^(19-20,22-25)^.

After a meticulous evaluation of responses, modifications suggested by the expert group were made, mainly revisions to simplify and clarify the initial version of the instrument without altering the proposed structure of each care indicator and the instrument as a whole.

Given the agreement on all indicators and their respective graded situations, as well as a CVI = 1, the content validity of the ICGP was ensured^(23-25)^. In its final version, it consisted of ten nursing care demand indicators with their respective conceptual definitions. For each indicator, four graded situations from 1 to 4 points were established, increasing according to nursing demand.

With the simultaneous application of the ICGP and Fugulin’s Patient Classification Instrument, convergent construct validity was obtained with a Spearman correlation coefficient above 0.5, considered a strong correlation^(21)^. The obtained value of 0.64 indicated that both instruments correlate in what they propose to measure: the classification of patients. The ICGP’s specificity could enhance the assessment of obstetric nursing by evaluating specific indicators such as “Breast Care” and “Family Support Network and Understanding of Health Status”. Evaluating these specific indicators is deemed essential for qualified obstetric nursing care, as pregnant and postpartum women have specific care needs during hospitalization.

The care indicators of the Pregnant and Postpartum Women Classification Instrument refer to specific situations of the gravid-puerperal period requiring specialized nursing assistance.

### Study limitations

A limitation was obtaining a homogeneous sample of obstetric patients amid changing profiles of obstetric patients in the study maternity ward. From November 2021, because of renovations in the intensive and semi-intensive neonatology unit, there was a reduction in obstetric hospitalization beds and a review of patient referrals in the region’s health system, leading to a decrease in complexity at the maternity ward where data collection occurred. Additionally, pandemic conditions prevented the collection of data for the simultaneous evaluation by different nurses and analysis of the ICGP’s inter rater reliability.

Further reliability studies of this instrument and its application in other settings that include high-risk pregnant and postpartum women are needed to assess its representativeness across all care categories.

### Contributions to Nursing, Health, or Public Policy

The main contribution is advancing research on the adequacy and creation of a specific instrument for classifying hospitalized pregnant and postpartum women, aiding in better nursing sizing through improved categorization of care needs in obstetric hospitalization wards.

The specificity of indicators related to the gravid-puerperal cycle could enhance nursing assessment based on the care demand of pregnant and postpartum women in management practice of patient classification.

## CONCLUSIONS

This study allowed for the development and availability of a specific instrument for classifying pregnant and postpartum patients in obstetric hospitalization units across the five care categories considered in national legislation for patient classification: minimal care, intermediate care, high-dependency care, semi-intensive care, and intensive care.

The instrument showed evidence of content validity and convergent construct validity. The specificity of indicators related to the gravid-puerperal cycle could enhance nursing assessment based on the care demand of pregnant and postpartum women in daily practice, for both operational care management and tactical nursing management in calculating nursing staff sizing.

Further studies are recommended to assess the construct validity and inter-rater reliability before implementing the instrument in obstetric hospitalization wards.

## Supplementary Material

0034-7167-reben-77-02-e20230401-Suppl01

0034-7167-reben-77-02-e20230401-Suppl02

0034-7167-reben-77-02-e20230401-Suppl03

## Data Availability

https://doi.org/10.25824/redu/LZIFAD
