# Supplementary material for: Validation of an instrument for patient classification to support obstetric nursing care
Source: Rev Bras Enferm. 2024 Jul 19;77(2):e20230401. doi: 10.1590/0034-7167-2023-0401 (PMC11259436; doi:10.1590/0034-7167-2023-0401)
Supplement: 0034-7167-reben-77-02-e20230401-Suppl01 [file 0034-7167-reben-77-02-e20230401-Suppl01.pdf]

Carimbo de data/hora	Endereço de e-mail	, benefícios previstos, pe	Completo do Particip	Contato Telefônico
9/22/2021 1:30:57	1	Sim		
12/8/2021 21:59:17	2	Sim		
1/27/2022 15:28:34	3	Sim		
1/27/2022 18:42:36	4	Sim		
1/28/2022 16:41:36	5	Sim		
1/31/2022 13:00:01	6	Sim		
2/2/2022 0:12:04	7	Sim		
2/2/2022 14:58:24	8	Sim		
2/3/2022 16:17:09	9	Sim		
2/3/2022 20:16:26	10	Sim		
2/9/2022 11:40:21	11	Sim		
2/25/2022 8:52:45	12	Sim		

Idade	Tempo de Experiência profissional
41	18 anos
45	25 anos
48 anos	26 anos
43	24 ANOS
28	4 anos
44	21 anos
33	Enfermeira há 7 anos
30	9 anos
43	19 anos
31	9/10 anos
42	20 anos
35	13 anos

Área e local de atuação profissional

Hositalar

Unidade de Internação

Obstetrícia

ENFERMARIA DE OBSTETRICIA

Maternidade e Centro Obstétrico - Hospital e Maternidade Madre Theodora

Enfermagem

Unidade de internação Obstétrica

Unidade de internação em obstetrícia - CAISM

Coordenação de Enfermagem - CAISM

Obstetrícia - CAISM Unicamp

Docência - Faculdade de Enfermagem da Unicamp

Coordenadora do Serviço de Enfermagem - Áreas Clínicas do AME de Limeira-SP, Docente de Curso de Graduação em

;raduação (Curso e Ano de Formaçãi	Especialização/ Resid
Enfermagem/ 2003	Sim
Enfemagem 2001	Sim
Enfermagem 1995	Sim
ENFERMAGEM 2007	Sim
Enfermeira - 2016	Sim
Enfermagem 1999	Não
Enfermagem - 2012	Sim
Enfermagem, 2012	Não
Enfermagem 2002	Sim
Enfermagem - 2012	Sim
Enfermagem - 2002	Sim
Enfermagem (2008)	Sim

Se sim , em qual área de formação?	Possui Mestrado ?
UTI, Mba em Gestão Hospitalar, Estética	Não
Ensino	Não
Obstetrícia, Saude Publica e Educação	Não
A EM GESTAÃO DE PESSOAS, ENFERMEGEM DO TRABALHO, ENFERMAGEM OBSTÉTR	Não
Enfermagem Obstétrica	Não
	Sim
Docência/Obstetricia	Não
	Não
Estomaterapia	Sim
Enfermagem em Neonatologia	Não
Pediatria, Estomaterapia, Oncologia e Avaliação de Tecnologias em Saúde	Sim
MBA em Gestão de Serviços de Saúde (FGV)	Sim

Se sim , qual o tema do seu Mestrado?

Relação entre uso de Uti e mortalidade materna

Validação de conteúdo, constructo e critério de instrumento para avaliação de impacto da doença

Validação de instrumento - Oncologia pediátrica

ão cultural e validação de instrumento de avaliação das experiências e da satisfação do paciente com o cuidado de enfe

Possui Doutorado ?
Não
Não
Não
Não
Não
Não
Não
Não
Sim
Não
Sim
Sim

Se sim , qual o tema do seu Doutorado?

Avaliação da responsividade de instrumento para avaliação de impacto da doença

Ambiente da prática profissional em Pediatria

ca de enfermagem e sua relação com a satisfação no trabalho, clima de segurança e intenção de permanecer por enferm

neiros do Estado de São Paulo.
