# Supplementary material for: Validation of an instrument for patient classification to support obstetric nursing care
Source: Rev Bras Enferm. 2024 Jul 19;77(2):e20230401. doi: 10.1590/0034-7167-2023-0401 (PMC11259436; doi:10.1590/0034-7167-2023-0401)

e

	Data	Leito	Indicador 1	2	3	4	5	6	7
1	11-Apr	102A	1	1	1	2	1	2	1
2	11-Apr	102C	1	1	1	1	1	2	1
3	11-Apr	107A	1	1	1	1	1	3	1
4	11-Apr	307A	1	1	1	1	1	1	1
5	13-Apr	102B	1	1	2	1	1	2	1
6	13-Apr	102C	1	1	1	1	1	2	1
7	13-Apr	105A	1	1	1	1	1	2	1
8	13-Apr	105B	1	1	1	1	1	1	1
9	13-Apr	107B	1	1	2	1	1	2	1
10	17-Apr	107A	1	1	1	1	1	1	2
11	17-Apr	106A	1	1	1	1	1	1	3
12	17-Apr	106C	1	1	1	1	1	1	2
13	17-Apr	108B	1	1	1	3	1	1	1
14	19-Apr	104A	2	1	1	1	2	1	1
15	19-Apr	104B	2	1	1	4	1	2	3
16	19-Apr	105A	1	2	1	1	2	1	1
17	19-Apr	106A	1	1	1	3	2	1	1
18	19-Apr	106B	2	1	1	1	2	1	1
19	19-Apr	106C	1	1	1	1	2	1	1
20	19-Apr	108A	2	1	1	1	2	1	1
21	19-Apr	108B	1	1	1	1	2	1	1
22	21-Apr	102A	2	1	2	1	1	2	2
23	21-Apr	102C	2	1	2	1	1	2	2
24	21-Apr	104A	2	1	2	1	1	2	1
25	21-Apr	104B	1	1	1	2	1	1	1
26	21-Apr	108A	1	1	2	2	1	1	1
27	21-Apr	108B	2	1	2	1	1	1	1
28	29-Apr	104B	1	1	1	1	1	2	1
29	29-Apr	105B	2	1	1	1	1	2	1
30	29-Apr	106A	1	1	1	1	1	3	1
31	29-Apr	106B	2	1	1	1	1	2	1
32	29-Apr	106C	2	1	1	3	1	2	1
33	29-Apr	107C	1	1	1	1	1	2	1
34	29-Apr	108B	1	1	1	1	1	1	1
35	3-May	101A	1	1	1	1	2	1	1
36	3-May	101B	1	1	1	1	1	1	1
37	3-May	105A	1	1	1	1	2	1	1
38	3-May	105B	1	1	1	1	1	1	1
39	3-May	106A	1	1	1	1	2	1	1
40	3-May	106B	1	1	1	1	2	1	1
41	3-May	106C	1	1	3	1	1	1	1
42	5-May	107A	1	1	1	1	1	2	2
43	5-May	107C	1	1	1	1	1	1	1
44	10-Jul	106A	1	1	1	1	1	1	1
45	10-Jul	106C	1	1	1	1	1	2	2
46	10-Jul	107A	1	1	1	1	1	1	1

47	10-Jul	107C	1	1	1	1	1	1	1
48	12-Jul	102A	1	1	1	1	1	1	1
49	12-Jul	102B	1	1	1	1	1	1	2
50	12-Jul	103A	1	1	1	1	1	2	2
51	12-Jul	103C	1	1	1	1	1	2	2
52	12-Jul	106A	1	1	1	1	1	1	1
53	12-Jul	106B	1	1	1	1	1	2	1
54	12-Jul	106C	2	1	1	4	2	2	3
55	12-Jul	107A	1	1	1	1	1	2	1
56	12-Jul	107C	1	1	1	1	1	2	1
57	12-Jul	108A	1	1	1	1	1	2	2
58	14-Jul	106A	1	1	1	1	1	1	1
59	14-Jul	106B	1	1	1	1	1	1	1
60	14-Jul	106C	1	1	1	1	1	2	1
61	14-Jul	107A	2	1	1	1	1	2	1
62	14-Jul	107B	1	1	1	1	1	2	1
63	14-Jul	107C	1	1	1	1	1	1	1
64	17-Jul	101A	1	1	1	1	1	2	2
65	17-Jul	102C	1	1	1	1	1	2	1
66	17-Jul	106A	1	1	2	1	1	1	1
67	17-Jul	106B	1	1	2	1	1	1	1
68	18-Jul	101A	2	1	1	1	1	1	1
69	18-Jul	102A	1	1	1	1	1	2	1
70	18-Jul	102C	1	1	1	1	1	3	1
71	18-Jul	103A	1	1	1	1	1	1	1
72	18-Jul	107A	1	1	1	1	1	1	1
73	18-Jul	107C	1	1	1	1	1	1	1
74	20-Jul	101A	2	1	1	2	1	2	1
75	20-Jul	101B	3	1	1	1	1	2	1
76	20-Jul	102A	1	1	1	1	1	2	2
77	20-Jul	102C	3	1	1	1	1	2	1
78	20-Jul	103A	1	1	1	1	1	2	2
79	20-Jul	103B	2	1	1	3	1	2	2
80	20-Jul	107A	1	1	2	1	1	2	1
81	20-Jul	107B	1	1	2	1	1	2	1
82	20-Jul	107C	1	1	1	1	1	2	2
83	22-Jul	101A	2	1	1	2	1	2	1
84	22-Jul	102A	1	1	1	1	1	2	1
85	22-Jul	102B	2	1	1	1	1	1	1
86	22-Jul	103A	1	1	1	1	1	1	1
87	22-Jul	103C	2	1	1	1	1	2	1
88	22-Jul	106A	1	1	2	1	1	1	1
89	22-Jul	107A	1	1	2	1	1	2	1
90	22-Jul	107B	1	1	1	1	1	1	1
91	22-Jul	107C	1	1	1	1	1	2	2
92	24-Jul	101A	1	1	1	1	1	1	1
93	24-Jul	102A	2	1	1	1	1	2	1

94	24-Jul	102C	2	1	1	1	1	1	1
95	24-Jul	103A	1	1	1	1	1	2	1
96	24-Jul	103B	2	1	1	1	1	2	1
97	24-Jul	103C	2	1	1	1	1	2	1
98	24-Jul	106A	1	1	1	1	1	2	1
99	24-Jul	106B	2	1	1	1	1	2	1
100	24-Jul	106C	1	1	1	1	1	1	1
101	26-Jul	102A	2	1	1	2	1	2	1
102	26-Jul	102C	1	1	1	1	1	1	1
103	26-Jul	105B	3	1	1	4	1	1	3
104	26-Jul	106C	1	1	1	1	1	1	1

8	9	10	Total	Classificação
3	2	1	15	Minimo
1	2	1	12	Minimo
1	1	1	12	Minimo
1	1	1	10	Minimo
1	1	1	12	Minimo
1	1	1	11	Minimo
1	1	1	11	Minimo
1	1	1	10	Minimo
1	1	1	12	Minimo
1	1	1	11	Minimo
1	1	1	12	Minimo
2	2	1	13	Minimo
1	2	1	13	Minimo
1	1	1	12	Minimo
1	1	1	17	Intermediario
1	1	1	12	Minimo
1	1	1	13	Minimo
1	1	1	12	Minimo
3	1	1	13	Minimo
1	1	1	12	Minimo
1	1	1	11	Minimo
2	2	1	16	Intermediario
2	2	1	16	Intermediario
1	1	1	13	Minimo
1	1	1	11	Minimo
1	1	1	12	Minimo
1	1	1	12	Minimo
1	1	1	11	Minimo
1	1	1	12	Minimo
1	1	1	12	Minimo
1	1	1	12	Minimo
1	1	1	14	Minimo
1	1	1	11	Minimo
1	1	1	10	Minimo
1	1	1	11	Minimo
1	1	1	10	Minimo
1	1	1	11	Minimo
2	2	1	12	Minimo
1	1	1	11	Minimo
1	1	1	11	Minimo
1	1	1	12	Minimo
1	1	1	12	Minimo
1	1	1	10	Minimo
1	1	1	10	Minimo
1	1	1	12	Minimo
1	2	1	11	Minimo

Classifi	
**De**	**Ate**
10	15
16	21
22	27
28	33
34	

1	1	1	10	Minimo
1	2	1	11	Minimo
1	1	1	11	Minimo
2	2	1	14	Minimo
2	2	1	14	Minimo
1	1	1	10	Minimo
1	1	1	11	Minimo
1	1	1	18	Intermediario
2	2	1	13	Minimo
1	1	1	11	Minimo
1	1	1	12	Minimo
1	1	1	10	Minimo
1	1	1	10	Minimo
1	1	1	11	Minimo
1	1	2	13	Minimo
1	1	1	11	Minimo
1	1	1	10	Minimo
1	1	1	12	Minimo
1	2	1	12	Minimo
1	1	1	11	Minimo
1	1	1	11	Minimo
1	1	1	11	Minimo
2	1	1	12	Minimo
2	2	2	15	Minimo
1	1	1	10	Minimo
1	1	1	10	Minimo
1	1	1	10	Minimo
1	1	2	14	Minimo
1	1	1	13	Minimo
2	2	1	14	Minimo
1	2	2	15	Minimo
1	1	1	12	Minimo
1	1	1	15	Minimo
1	1	1	12	Minimo
1	1	1	12	Minimo
2	2	1	14	Minimo
1	1	1	13	Minimo
1	1	1	11	Minimo
1	1	2	12	Minimo
1	1	1	10	Minimo
2	2	2	15	Minimo
1	1	1	11	Minimo
1	1	1	12	Minimo
1	1	1	10	Minimo
2	2	1	14	Minimo
1	1	1	10	Minimo
1	1	1	12	Minimo

1	1	1	11	Minimo
1	1	1	11	Minimo
1	1	1	12	Minimo
1	1	1	12	Minimo
1	1	1	11	Minimo
1	1	1	12	Minimo
1	1	1	10	Minimo
1	1	1	13	Minimo
3	1	1	12	Minimo
1	1	1	17	Intermediario
1	2	1	11	Minimo

icação
Minimo
Intermediario
Alta Dependencia
Semi intensivo
Intensivo

			Indicador						
	Data	Leito	1	2	3	4	5	6	7
35	3/5	101A	2	1	2	1	1	2	1
36	3/5	101B	4	1	2	2	1	2	1
37	3/5	105A	2	1	2	1	1	2	1
38	3/5	105B	1	1	2	1	1	1	1
39	3/5	106A	2	1	1	1	1	2	2
40	3/5	106B	2	1	2	3	1	2	1
41	3/5	106C	1	1	2	1	1	1	1
42	5/5	107A	2	1	1	1	1	1	2
43	5/5	107C	1	1	1	1	1	1	1
44	10/7	106A	1	1	1	1	1	2	2
45	10/7	106C	1	1	2	1	1	2	1
46	10/7	107A	1	1	2	1	1	1	1
47	10/7	107C	2	1	2	1	1	1	1
48	12/7	102A	2	1	1	4	2	2	3
49	12/7	102B	1	1	2	1	2	2	1
50	12/7	103A	1	1	2	1	1	2	1
51	12/7	103C	1	1	2	1	1	1	1
52	12/7	106A	1	1	2	1	1	1	1
53	12/7	106B	1	1	2	1	1	1	1
54	12/7	106C	1	1	1	3	1	2	1
55	12/7	107A	2	1	1	1	1	2	1
56	12/7	107C	1	1	1	1	1	2	1
57	12/7	108A	1	1	2	1	1	1	1
58	14/7	106A	2	1	1	1	1	2	2
59	14/7	106B	1	1	1	1	1	1	1
60	14/7	106C	1	1	2	1	1	1	1
61	14/7	107A	1	1	2	1	1	1	1
62	14/7	107B	2	1	1	1	1	2	1
63	14/7	107C	2	1	1	1	1	2	1
64	17/7	101A	2	1	1	1	1	2	1
65	17/7	102C	2	1	2	1	1	1	1
66	17/7	106A	1	1	2	1	1	1	1
67	17/7	106B	1	1	2	1	1	1	1
68	18/7	101A	2	1	1	1	1	2	1
69	18/7	102A	2	1	2	1	1	2	1
70	18/7	102C	1	1	1	1	1	2	2
71	18/7	103A	2	1	1	1	1	2	1
72	18/7	107A	2	1	2	1	1	2	2
73	18/7	107C	2	1	1	1	1	2	2
74	20/7	101A	1	1	2	1	1	2	1
75	20/7	101B	1	1	2	1	1	2	1
76	20/7	102A	1	1	2	1	1	2	2

77	20/7	102C	2	1	1	2	1	2	1
78	20/7	103A	1	1	1	1	1	2	1
79	20/7	103B	2	1	1	1	1	1	1
80	20/7	107A	2	1	1	1	1	1	1
81	20/7	107B	2	1	1	1	1	1	2
82	20/7	107C	2	1	1	1	1	2	1
83	22/7	101A	1	1	2	1	1	2	1
84	22/7	102A	1	1	1	1	1	1	1
85	22/7	102B	1	1	1	1	1	2	2
86	22/7	103A	1	1	2	1	1	1	1
87	22/7	103C	2	1	2	2	1	2	2
88	22/7	106A	2	1	1	1	1	1	1
89	22/7	107A	2	1	2	1	1	2	1
90	22/7	107B	2	1	2	1	1	2	1
91	22/7	107C	2	1	2	1	1	2	2
92	24/7	101A	2	1	2	1	1	2	2
93	24/7	102A	2	1	2	1	1	2	2
94	24/7	102C	1	1	2	1	1	2	1
95	24/7	103A	2	1	1	1	1	2	1
96	24/7	103B	1	1	2	1	1	1	1
97	24/7	103C	2	1	1	2	1	2	1
98	24/7	106A	1	1	1	1	1	1	1
99	24/7	106B	3	1	1	4	1	1	3
100	24/7	106C	1	1	1	1	1	1	1
101	26/7	102A	2	1	2	1	1	2	1
102	26/7	102C	1	1	1	3	1	1	1
103	26/7	105B	1	1	2	3	1	1	1
104	26/7	106C	2	1	2	1	1	1	1
							1		

8	9	10	Total	Classificação
1	1	1	13	Minimo
1	1	1	16	Intermediario
1	1	1	13	Minimo
1	1	1	11	Minimo
1	1	1	13	Minimo
1	2	1	16	Intermediario
1	1	1	11	Minimo
1	1	1	12	Minimo
1	1	1	10	Minimo
2	2	1	14	Minimo
2	1	1	13	Minimo
1	1	1	11	Minimo
1	1	1	12	Minimo
1	1	1	18	Intermediario
1	2	1	14	Minimo
1	1	1	12	Minimo
1	1	1	11	Minimo
1	1	1	11	Minimo
1	1	1	11	Minimo
1	1	1	13	Minimo
1	1	1	12	Minimo
1	1	1	11	Minimo
1	1	1	11	Minimo
1	1	1	13	Minimo
1	1	1	10	Minimo
1	1	1	11	Minimo
1	1	1	11	Minimo
2	2	1	14	Minimo
2	2	1	14	Minimo
1	2	2	14	Minimo
1	1	1	12	Minimo
1	1	1	11	Minimo
1	1	1	11	Minimo
1	1	1	12	Minimo
1	1	1	13	Minimo
1	1	1	12	Minimo
1	1	2	13	Minimo
1	1	1	14	Minimo
1	1	1	13	Minimo
1	1	1	12	Minimo
1	1	1	12	Minimo
2	2	1	15	Minimo

Classifi

Data	Minimo
11-Apr	3
13-Apr	5
17-Apr	4
19-Apr	5
21-Apr	6
29-Apr	6
ABRIL	29
3-May	5
5-May	2
MAIO	7
10-Jul	4
12-Jul	9
14-Jul	6
17-Jul	4
18-Jul	6
20-Jul	9
22-Jul	8
24-Jul	6
26-Jul	4
JULHO	56
9-Aug	10
21-Aug	6

1	1	1	13	Minimo
1	1	1	11	Minimo
1	1	2	12	Minimo
1	1	1	11	Minimo
2	2	2	15	Minimo
1	1	1	12	Minimo
1	1	1	12	Minimo
1	1	1	10	Minimo
2	2	1	14	Minimo
1	1	1	11	Minimo
2	1	1	16	Intermediario
1	1	1	11	Minimo
2	1	1	14	Minimo
2	1	1	14	Minimo
1	1	1	14	Minimo
2	2	1	16	Intermediario
2	2	1	16	Intermediario
1	1	1	12	Minimo
1	2	1	13	Minimo
1	1	1	11	Minimo
1	1	1	13	Minimo
3	1	1	12	Minimo
1	1	1	17	Intermediario
1	1	1	10	Minimo
1	1	1	13	Minimo
1	1	1	12	Minimo
1	1	1	13	Minimo
1	1	1	12	Minimo

AGOSTO	16
TOTAL	108

icação

Intermediario	Alta Dependencia	Semi Intensivo	*ntensivo*	
1	0	0	0	4
0	0	0	0	5
0	0	0	0	4
3	0	0	0	8
0	0	0	0	6
1	0	0	0	7
5	0	0	0	34
2	0	0	0	7
0	0	0	0	2
2	0	0	0	9
0	0	0	0	4
1	0	0	0	10
0	0	0	0	6
0	0	0	0	4
0		0	0	6
0	0	0	0	9
1	0	0	0	9
3	0	0	0	9
0	0	0	0	4
5	0	0	0	61
0	0	0	0	10
0	0	0	0	6

0	0	0	0	16
12	0	0	0	120

	Data	Leito	1	2	3	4	5	6	7	8
1	11-Apr	102A	1	1	2	2	2	1	1	1
2	11-Apr	102C	1	1	2	1	1	1	1	1
3	11-Apr	107A	1	1	2	1	1	1	1	1
4	11-Apr	307A	1	1	3	1	1	1	1	1
5	13-Apr	102B	1	1	3	1	1	1	1	1
6	13-Apr	102C	1	1	2	1	2	1	2	1
7	13-Apr	105A	1	1	2	1	1	1	1	1
8	13-Apr	105B	1	1	2	1	1	1	1	1
9	13-Apr	107B	1	1	3	1	1	1	1	1
10	17-Apr	107A	1	1	1	1	1	1	1	1
11	17-Apr	106A	1	1	1	1	1	1	1	1
12	17-Apr	106C	1	1	1	1	1	1	1	1
13	17-Apr	108B	1	1	1	1	2	1	2	1
14	19-Apr	104A	1	1	3	1	1	1	1	1
15	19-Apr	104B	1	1	2	1	4	2	4	3
16	19-Apr	105A	1	1	3	1	2	2	2	2
17	19-Apr	106A	1	1	3	1	1	1	1	1
18	19-Apr	106B	1	1	2	1	1	1	1	1
19	19-Apr	106C	1	1	3	1	2	1	1	1
20	19-Apr	108A	1	1	2	1	1	1	1	1
21	19-Apr	108B	1	1	3	1	1	1	1	1
22	21-Apr	102A	1	1	1	1	1	1	1	1
23	21-Apr	102C	1	1	3	1	1	1	1	1
24	21-Apr	104A	1	1	2	1	1	1	1	1
25	21-Apr	104B	1	1	3	1	1	1	1	1
26	21-Apr	108A	1	1	3	1	1	1	1	1
27	21-Apr	108B	1	1	2	1	1	1	1	1
28	29-Apr	104B	1	1	2	1	1	1	1	1
29	29-Apr	105B	1	1	3	1	1	1	1	1
30	29-Apr	106A	1	1	2	1	1	1	1	1
31	29-Apr	106B	1	1	4	1	1	1	1	1
32	29-Apr	106C	1	1	2	1	1	1	1	1
33	29-Apr	107C	1	1	2	1	1	1	1	1
34	29-Apr	108B	1	1	3	1	1	1	1	1
35	3-May	101A	1	1	3	1	1	1	1	1
36	3-May	101B	1	1	3	1	1	1	1	1
37	3-May	105A	1	1	3	1	1	1	1	1
38	3-May	105B	1	1	3	1	1	1	1	1
39	3-May	106A	1	1	2	1	1	1	2	1
40	3-May	106B	1	1	3	1	1	1	1	1
41	3-May	106C	1	1	3	1	1	1	1	1
42	5-May	107A	1	1	2	1	1	1	1	1
43	5-May	107C	1	1	2	1	1	1	1	1
44	10-Jul	106A	1	1	2	1	1	1	1	1
45	10-Jul	106C	1	1	3	1	1	1	1	1

46	10-Jul	107A	1	1	3	1	1	1	1	1
47	10-Jul	107C	1	1	3	1	1	1	1	1
48	12-Jul	102A	1	1	2	1	4	1	4	3
49	12-Jul	102B	1	1	3	1	1	1	1	1
50	12-Jul	103A	1	1	3	1	1	1	2	1
51	12-Jul	103C	1	1	3	1	1	1	2	1
52	12-Jul	106A	1	1	3	1	1	1	1	1
53	12-Jul	106B	1	1	3	1	1	1	1	1
54	12-Jul	106C	1	1	2	1	1	1	1	1
55	12-Jul	107A	1	1	2	1	1	1	1	1
56	12-Jul	107C	1	1	2	1	1	1	1	1
57	12-Jul	108A	1	1	3	1	1	1	1	1
58	14-Jul	106A	1	1	2	1	1	1	1	1
59	14-Jul	106B	1	1	2	1	1	1	1	1
60	14-Jul	106C	1	1	2	1	1	1	1	1
61	14-Jul	107A	1	1	2	1	1	1	1	1
62	14-Jul	107B	1	1	2	1	1	1	1	1
63	14-Jul	107C	1	1	2	1	1	1	1	1
64	17-Jul	101A	1	1	3	1	1	1	1	1
65	17-Jul	102C	1	1	3	1	1	1	1	1
66	17-Jul	106A	1	1	3	1	1	1	1	1
67	17-Jul	106B	1	1	3	1	1	1	1	1
68	18-Jul	101A	1	1	2	1	1	1	1	1
69	18-Jul	102A	1	1	3	1	1	1	1	1
70	18-Jul	102C	1	1	2	1	1	1	1	1
71	18-Jul	103A	1	1	2	1	1	1	1	1
72	18-Jul	107A	1	1	3	1	2	1	1	1
73	18-Jul	107C	1	1	2	1	1	1	1	1
74	20-Jul	101A	1	1	3	1	1	1	1	1
75	20-Jul	101B	1	1	3	1	1	1	1	1
76	20-Jul	102A	1	1	3	1	1	1	1	1
77	20-Jul	102C	1	1	2	1	1	1	1	1
78	20-Jul	103A	1	1	2	1	1	1	1	1
79	20-Jul	103B	1	1	2	1	1	1	1	1
80	20-Jul	107A	1	1	2	1	1	1	1	1
81	20-Jul	107B	1	1	2	1	1	1	1	1
82	20-Jul	107C	1	1	2	1	1	1	1	1
83	22-Jul	101A	1	1	3	1	1	1	1	1
84	22-Jul	102A	1	1	2	1	1	1	1	1
85	22-Jul	102B	1	1	2	1	1	1	1	1
86	22-Jul	103A	1	1	3	1	1	1	1	1
87	22-Jul	103C	1	1	3	1	2	1	1	1
88	22-Jul	106A	1	1	2	1	1	1	1	1
89	22-Jul	107A	1	1	3	1	1	1	1	1
90	22-Jul	107B	1	1	3	1	1	1	1	1
91	22-Jul	107C	1	1	3	1	1	1	1	1
92	24-Jul	101A	1	1	3	1	1	1	1	1

93	24-Jul	102A	1	1	2	1	1	1	1	1
94	24-Jul	102C	1	1	3	1	1	1	1	1
95	24-Jul	103A	1	1	2	1	1	1	1	1
96	24-Jul	103B	1	1	2	1	1	1	1	1
97	24-Jul	103C	1	1	2	1	1	1	1	1
98	24-Jul	106A	1	1	2	1	1	1	1	1
99	24-Jul	106B	1	1	3	1	1	1	1	1
100	24-Jul	106C	1	1	3	1	1	1	1	1
101	26-Jul	102A	1	1	3	1	1	1	1	1
102	26-Jul	102C	1	1	2	1	2	1	2	1
103	26-Jul	105B	1	1	3	1	1	1	2	1
104	26-Jul	106C	1	1	3	1	1	1	1	1
105	9-Aug	102A	1	1	2	1	1	1	1	1
106	9-Aug	102B	1	1	3	1	2	1	1	1
107	9-Aug	102C	1	1	2	1	1	1	1	1
108	9-Aug	103B	1	1	3	1	1	1	1	1
109	9-Aug	103C	1	1	2	1	1	1	1	2
110	9-Aug	106A	1	1	3	1	1	1	1	1
111	9-Aug	106C	1	1	2	1	1	1	1	1
112	9-Aug	107A	1	1	2	1	1	1	1	1
113	9-Aug	107C	1	1	3	1	1	1	1	1
114	9-Aug	108A	1	1	3	1	2	1	1	1
115	21-Aug	102A	1	1	3	1	1	1	1	1
116	21-Aug	102C	1	1	2	1	1	1	1	1
117	21-Aug	106A	1	1	2	1	1	1	1	1
118	21-Aug	107A	1	1	3	1	1	1	1	1
119	21-Aug	107B	1	1	3	1	1	1	2	1
120	21-Aug	107C	1	1	3	1	1	1	1	1

9	Total	Classificação
2	13	Minimo
2	11	Minimo
2	11	Minimo
1	11	Minimo
1	11	Minimo
2	13	Minimo
1	10	Minimo
1	10	Minimo
1	11	Minimo
2	10	Minimo
3	11	Minimo
2	10	Minimo
1	11	Minimo
2	12	Minimo
2	20	Intermediario
2	16	Intermediario
2	12	Minimo
2	11	Minimo
2	13	Minimo
2	11	Minimo
2	12	Minimo
2	10	Minimo
2	12	Minimo
2	11	Minimo
1	11	Minimo
1	11	Minimo
2	11	Minimo
2	11	Minimo
2	12	Minimo
2	11	Minimo
3	14	Minimo
1	10	Minimo
1	10	Minimo
1	11	Minimo
2	12	Minimo
2	12	Minimo
2	12	Minimo
1	11	Minimo
2	12	Minimo
2	12	Minimo
1	11	Minimo
1	10	Minimo
1	10	Minimo
2	11	Minimo
2	12	Minimo

Classificação		
De	Ate	
9	14	Minimo
15	20	Intermediario
21	26	Alta Dependencia
27	31	Semi intensivo
32		Intensivo

1	11	Minimo
1	11	Minimo
2	19	Intermediario
2	12	Minimo
2	13	Minimo
2	13	Minimo
1	11	Minimo
1	11	Minimo
2	11	Minimo
2	11	Minimo
2	11	Minimo
1	11	Minimo
1	10	Minimo
1	10	Minimo
1	10	Minimo
1	10	Minimo
1	10	Minimo
1	10	Minimo
1	11	Minimo
1	11	Minimo
1	11	Minimo
1	11	Minimo
2	11	Minimo
2	12	Minimo
2	11	Minimo
2	11	Minimo
2	13	Minimo
2	11	Minimo
2	12	Minimo
2	12	Minimo
2	12	Minimo
2	11	Minimo
2	11	Minimo
1	10	Minimo
1	10	Minimo
1	10	Minimo
2	11	Minimo
2	12	Minimo
1	10	Minimo
2	11	Minimo
1	11	Minimo
2	13	Minimo
1	10	Minimo
2	12	Minimo
2	12	Minimo
2	12	Minimo
2	12	Minimo

2	11	Minimo
1	11	Minimo
1	10	Minimo
1	10	Minimo
1	10	Minimo
1	10	Minimo
2	12	Minimo
2	12	Minimo
2	12	Minimo
1	12	Minimo
1	12	Minimo
1	11	Minimo
2	11	Minimo
2	13	Minimo
2	11	Minimo
1	11	Minimo
1	11	Minimo
1	11	Minimo
2	11	Minimo
1	10	Minimo
1	11	Minimo
2	13	Minimo
2	12	Minimo
1	10	Minimo
1	10	Minimo
2	12	Minimo
2	13	Minimo
2	12	Minimo

	ICGP	ICGP			Fugulin	Fugulin	
Data	Minimo	Intermediario			Minimo	Intermediario	
11/4	3	1	4		4	0	4
13/4	5	0	5		5	0	5
17/4	4	0	4		4	0	4
19/4	5	3	8		6	2	8
21/4	6	0	6		6	0	6
29/4	6	1	7		7	0	7
ABRIL	29	5	34		32	2	34
3/5	5	2	7		7	0	7
5/5	2	0	2		2	0	2
MAIO	7	2	9		9	0	9
10/7	4	0	4		4	0	4
12/7	9	1	10		9	1	10
14/7	6	0	6		6	0	6
17/7	4	0	4		4	0	4
18/7	6	0	6		6	0	6
20/7	9	0	9		9	0	9
22/7	8	1	9		9	0	9
24/7	6	3	9		9	0	9
26/7	4	0	4		4	0	4
JULHO	56	5	61		60	1	61
9/8	10	0	10		10	0	10
21/8	6	0	6		6	0	6
AGOSTO	16	0	16		16	0	16
TOTAL	108	12	120		117	3	120
	90.0%	10.0%	120		97.5%	2.5%	1.00

Data	ICGP Minimo	Fugulin Minimo	ICGP Intermediario	Fugulin Intermediario
ABRIL	29	32	5	2
MAIO	7	9	2	0
JULHO	56	60	5	1
AGOSTO	16	16	0	0
TOTAL	108	117	12	3

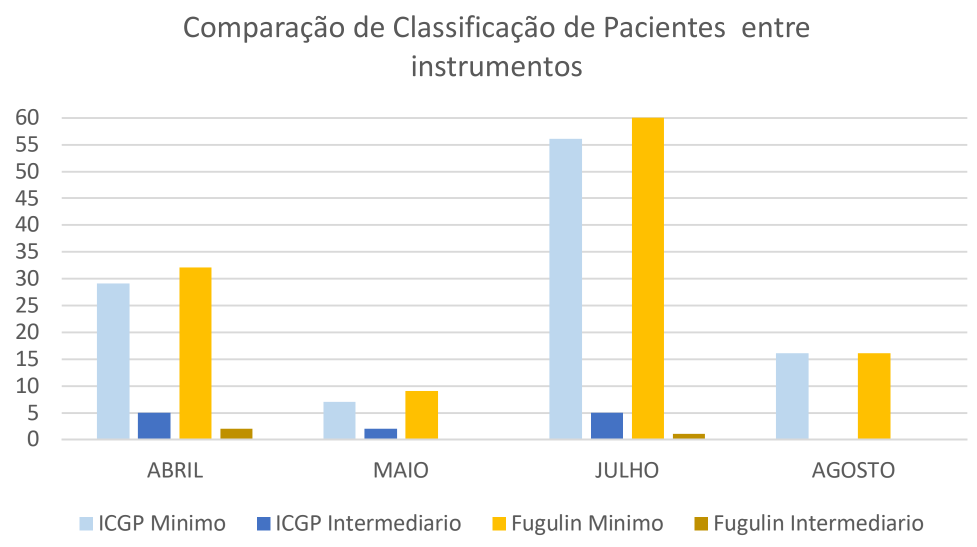

Supplement: 0034-7167-reben-77-02-e20230401-Suppl02 [file 0034-7167-reben-77-02-e20230401-Suppl02.pdf]
