# Supplementary material for: Validation of an instrument for patient classification to support obstetric nursing care
Source: Rev Bras Enferm. 2024 Jul 19;77(2):e20230401. doi: 10.1590/0034-7167-2023-0401 (PMC11259436; doi:10.1590/0034-7167-2023-0401)
Supplement: 0034-7167-reben-77-02-e20230401-Suppl03 [file 0034-7167-reben-77-02-e20230401-Suppl03.pdf]

- O indicador proposto é:

Relevante e representativo na avaliação da demanda de cuidados de enfermagem
Relevante e representativo na avaliação da demanda de cuidados de enfermagem
Relevante e representativo na avaliação da demanda de cuidados de enfermagem
Relevante e representativo na avaliação da demanda de cuidados de enfermagem
Relevante e representativo na avaliação da demanda de cuidados de enfermagem
Relevante e representativo na avaliação da demanda de cuidados de enfermagem
Precisa de pequena revisão para ser relevante ou apresentar clareza na avaliação da demanda de cuidados de enf
Relevante e representativo na avaliação da demanda de cuidados de enfermagem
Relevante e representativo na avaliação da demanda de cuidados de enfermagem
Precisa de pequena revisão para ser relevante ou apresentar clareza na avaliação da demanda de cuidados de enf
Precisa de pequena revisão para ser relevante ou apresentar clareza na avaliação da demanda de cuidados de enf
Relevante e representativo na avaliação da demanda de cuidados de enfermagem

As situações graduada	Sugestões
Sim	Nenhuma
Sim	Na
Sim	Considerar em alguns
Sim	NÃO TENHO SUGEST
Sim	.
Sim	Nenhuma
Sim	Acredito que os itens 3
Sim	Não há
Não	O item 2 - " Orientada
Sim	Tentar separar um pou
Sim	Substituir poliqueixosa
Sim	1-Orientada em tempo

- O indicador proposto é:

Precisa de pequena revisão para ser relevante ou apresentar clareza na avaliação da demanda de cuidados de enferma

Relevante e representativo na avaliação da demanda de cuidados de enfermagem

Relevante e representativo na avaliação da demanda de cuidados de enfermagem

Relevante e representativo na avaliação da demanda de cuidados de enfermagem

Precisa de pequena revisão para ser relevante ou apresentar clareza na avaliação da demanda de cuidados de enferma

Relevante e representativo na avaliação da demanda de cuidados de enfermagem

Relevante e representativo na avaliação da demanda de cuidados de enfermagem

Relevante e representativo na avaliação da demanda de cuidados de enfermagem

Relevante e representativo na avaliação da demanda de cuidados de enfermagem

Relevante e representativo na avaliação da demanda de cuidados de enfermagem

Precisa de pequena revisão para ser relevante ou apresentar clareza na avaliação da demanda de cuidados de enferma

Relevante e representativo na avaliação da demanda de cuidados de enfermagem

As situações graduada	Sugestões	- O indicador proposto	As situações graduada	Sugestões
Sim	Descrever o tipo de de	Relevante e representa	Sim	Nenhuma
Sim	Na	Relevante e representa	Sim	Na
Sim	NDN	Relevante e representa	Sim	NDN
Sim	NÃO TENHO SUGEST	Relevante e representa	Sim	FIQUEI EM DUVIDA S
Sim	.	Relevante e representa	Sim	.
Sim	Nenhuma	Relevante e representa	Sim	Nenhuma
Sim	Estive pensando no tip	Relevante e representa	Sim	Não há
Sim	Não há	Relevante e representa	Sim	Não há
Sim	Não vejo a questão da	Relevante e representa	Sim	Entendo que o item é i
Sim	sem sugestões	Relevante e representa	Sim	Acho que ficaram muit
Sim	Substituir traqueostomi	Relevante e representa	Sim	Nada a acrescentar.
Sim	II- Suporte respiratório:	Relevante e representa	Sim	III-Intervalo de aferiçã

- O indicador proposto	As situações graduada	Sugestões	- O indicador proposto	As situações graduada
Relevante e representa	Sim	Nenhuma⁸	Relevante e representa	Sim
Relevante e representa	Sim	Na	Relevante e representa	Sim
Relevante e representa	Sim	NDN	Relevante e representa	Sim
Relevante e representa	Sim	NÃO TENHO	Relevante e representa	Sim
Relevante e representa	Sim	Entre os itens 2/3 pode	Relevante e representa	Sim
Relevante e representa	Sim	Nenhuma	Relevante e representa	Sim
Relevante e representa	Sim	Talvez descrever este	Relevante e representa	Sim
Relevante e representa	Sim	Não há	Relevante e representa	Sim
Relevante e representa	Sim	sem sugestão	Relevante e representa	Sim
Relevante e representa	Sim	nenhuma	Relevante e representa	Sim
Precisa de pequena re	Sim	Em todos os níveis sub	Precisa de pequena re	Sim
Relevante e representa	Sim	Sem sugestões.	Relevante e representa	Sim

Sugestões	- O indicador proposto	As situações graduadas	Sugestões	- O indicador proposto
Colocaria náuseas e vó	Relevante e representa	Sim	Nenhuma	Relevante e representa
Na	Relevante e representa	Sim	Na	Relevante e representa
NDN	Relevante e representa	Sim	NDN	Relevante e representa
NÃO TENHO	Relevante e representa	Sim	NÃO TENHO	Relevante e representa
.	Relevante e representa	Sim	.	Relevante e representa
Nenhuma	Relevante e representa	Sim	Nenhuma	Relevante e representa
Não há	Relevante e representa	Sim	Talvez colocar a intram	Relevante e representa
Não há	Relevante e representa	Sim	Não há	Relevante e representa
sem sugestão	Relevante e representa	Sim	Sugiro a inclusão do su	Relevante e representa
nenhuma	Relevante e representa	Sim	A minha dúvida é se a	Relevante e representa
Excluiria da expressão	Relevante e representa	Sim	Nada a acrescentar	Precisa de pequena rev
V- Suporte alimentar e	Relevante e representa	Sim	Sem sugestões.	Precisa de pequena rev

As situações graduada	Sugestões	- O indicador proposto	As situações graduada	Sugestões
Sim	Não mencionaria a nor	Relevante e representa	Sim	Nenhuma
Sim	Na	Relevante e representa	Sim	Na
Sim	NDN	Relevante e representa	Sim	NDN
Sim	NÃO TENHO	Relevante e representa	Sim	NÃO TENHO
Sim	.	Relevante e representa	Sim	Poderia acrescentar a
Sim	Nenhuma	Relevante e representa	Sim	Nenhuma
Sim	Não há	Relevante e representa	Sim	Não há
Sim	Não há	Relevante e representa	Sim	Não há
Sim	Sugiro alterar a grafia	Relevante e representa	Sim	sem sugestão
Sim	nenhuma	Relevante e representa	Sim	nenhuma
Sim	Substituir " WC" por "b	Precisa de pequena re	Sim	Substituir "lubrificação'
Sim	Os itens contemplam c	Relevante e representa	Sim	Sem sugestões.

- O indicador proposto	As situações graduada	Sugestões	- O indicador proposto	As situações graduada
Precisa de pequena re	Sim	Não consegui pensar e	Relevante e representa	Sim
Relevante e representa	Sim	Na	Relevante e representa	Sim
Relevante e representa	Sim	NDN	Relevante e representa	Sim
Relevante e representa	Sim	NÃO TENHO	Relevante e representa	Sim
Relevante e representa	Sim	.	Relevante e representa	Sim
Relevante e representa	Sim	Nenhuma	Relevante e representa	Sim
Relevante e representa	Sim	Não há	Relevante e representa	Sim
Relevante e representa	Sim	Não há	Relevante e representa	Sim
Relevante e representa	Sim	sem sugestão	Relevante e representa	Sim
Relevante e representa	Sim	No 3 e 4 acho que pod	Relevante e representa	Sim
Relevante e representa	Sim	Nada a acrescentar.	Relevante e representa	Sim
Relevante e representa	Sim	4-Mamas com sinais d	Relevante e representa	Sim

Sugestões	Sugestões Gerais sobre o Instrumento
Nenhuma	Opções esclarecedoras ao realizar a leitura, apenas necessita, a meu ver, de pequenos ajust
Na	Instrumento bem formulado
NDN	
NÃO TENHO	
.	
Nenhuma	Nenhuma
Não há	
Não há	Instrumento muito bem construído, será de grande ajuda na assistência!
Sugiro a inclusão, talve	O instrumento me parece breve e prático. Gostaria de entender como seria usado os escores
Deixar claro no 4 que r	Muito bom!!
Nada a acrescentar	
Pequenas sugestões q	O item VII pode requerer revisão quanto à clareza para avaliação. No item X fiz uma sugestã

es para chegar mais próximo do ótimo

na prática clínica diária do enfermeiro.

o quanto à descrição que pode requerer revisão gramatical para assegurar clareza e objetividade à categoria de resp

osta. As demais sugestões são pequenas revisões, verifique se há sentido para os pesquisadores e/ou espec

cialistas.

Alternativa	I	II	III	IV	V	VI	VII
							Indicado[illegible]
**1.** Não relevante ou não apresenta clareza para avaliação da demanda de cuidados de enfermagem	0	0	0	0	0	0	0
**2.** Precisa de grande revisão para ser relevante ou apresentar clareza na avaliação da demanda de cuidados de enfermagem	0	0	0	0	0	0	0
**3.**Precisa de pequena revisão para ser relevante ou apresentar clareza na avaliação da demanda de cuidados de enfermagem	3	3	0	1	1	0	2
**4.**Relevante e representativo na avaliação da demanda de cuidados de enfermagem	9	9	12	11	11	12	10
**Índice de Validade de Conteúdo (IVC)**	1.0	1.0	1.0	1.0	1.0	1.0	1.0

As situações graduadas estão organizadas em ordem crescente quanto à demanda de cuidados de enf[illegible]

	I	II	III	IV	V	VI
						Indicado[illegible]
Sim	11	12	12	12	12	12
Não	1	0	0	0	0	0

res

VIII	IX	X
0	0	0
0	0	0
1	1	0
11	11	12
1.0	1.0	1.0

ermagem?

res

VII	VIII	IX	X
12	12	12	12
0	0	0	0

Indicador 1

Considerar em alguns momentos as alterações hormonais do puerperio como agravantes e
NÃO TENHO SUGESTÕES
.
Nenhuma
Acredito que os itens 3 e 4 possuem ampla abrangência na avaliação do estado mental.
Não há
O item 2 - " Orientada e/ou pouco ansiosa ou queixosa", não entendo que seja "e/ou", visto
Tentar separar um pouco do estado mental do estado comportamental
Substituir poliqueixosa por "múltiplas queixas"
1-Orientada em tempo, espaço e pessoa, tranquila. 2-Orientada e/ou pouco ansiosa ou que

Indicador 2

Descrever o tipo de desobstrução intencionada na pergunta. Sugiro ao invés desta ação, refletir sobre o uso intermitente de oxigênio
Na
NDN
NÃO TENHO SUGESTÕES
.
Nenhuma
Estive pensando no tipo de auxílio/orientação na desobstrução??, bem como no item 4 - as traqueostomizadas, podendo estar com v
Não há
Não vejo a questão da desobstrução de vias aéreas uma situação importante para ser pontuada no instrumento. No entanto, se as pe
sem sugestões
Substituir traqueostomizada por "com traqueostomia"
II- Suporte respiratório: Necessidade da puérpera ou gestante de auxílio para desobstrução de vias aéreas e/ou suplementação da ox

Indicador 3

Nenhuma
Não há
Na
NDN
FIQUEI EM DUVIDA SE EDEMA É CONSIDERADO COMO UMA EVIDENCIA DE PADRÕE
.
Entendo que o item é importante e representa uma demanda significativa para a enfermage
Acho que ficaram muitas informações para um só tópico; eu colocaria nesse a questão dos
Nada a acrescentar.
III-Intervalo de aferição de controles: Necessidade de observação e controle de dados como

Indicador 4

Nenhuma[8]
Na
NDN
NÃO TENHO
Entre os itens 2/3 poderia acrescentar a paciente que necessita de auxílio para as atividades como ba
Nenhuma

Talvez descrever este item como atividade diária, devêssemos incluir o auxílio na alimentação, como
Não há
sem sugestão
nenhuma
Em todos os níveis substituir "se banha" por higiene corporal
Sem sugestões.

Indicador 5

Não há
Colocaria náuseas e vômitos no número 2, visto a demanda de assistência não ser tão prol
Na
NDN
NÃO TENHO
.
Nenhuma
sem sugestão
nenhuma
Excluiria da expressão "sem auxílio da enfermagem" o termo "da enfermagem", pois mesmo
V- Suporte alimentar e hídrico: Necessidade de auxílio/ cuidados para ingerir alimentos e/ou

Indicador 6

Nenhuma
Na
NDN
NÃO TENHO
.
Nenhuma
Talvez colocar a intramuscular junto com a endovenosa intermitente
Não há
Sugiro a inclusão do subcutâneo, principalmente em relação as pacientes diabéticas ou em
A minha dúvida é se a via intramuscular entra no cuidado 1 mesmo?! Para mim entraria no
Nada a acrescentar
Sem sugestões.

Indicador 7

Não há
Não mencionaria a normalidade ou não da frequência urinária e intestinal, visto não impacta
Na
NDN
NÃO TENHO
.
Nenhuma
Sugiro alterar a grafia de ostomias para "estomias" conforme https://sobest.com.br/estomias
nenhuma
Substituir " WC" por "banheiro" e "ostomias" por "estomias"
Os itens contemplam ordem crescente quanto à demanda de cuidados. Só não estou certa

Indicador 8

Não há
Nenhuma
Na
NDN
NÃO TENHO
Poderia acrescentar a parte do períneo, períneo íntegro, com laceração que necessite de cu
Nenhuma
sem sugestão
nenhuma
Substituir "lubrificação" por "hidratação". Acrescentar no item 3 - superior a 15 minutos ou m
Sem sugestões.

Indicador 9

ão há
Não consegui pensar em tempos de assistência de enfermagem diferentes para itens 1 e 2.
Na
NDN
NÃO TENHO
.
Nenhuma
sem sugestão
No 3 e 4 acho que poderia colocar orientação pela enfermagem sobre massagem e ordenha
Nada a acrescentar.
4-Mamas com sinais de infecção ou reação hiperálgica ou fissura mamária, com necessidad

Indicador X

Nenhuma
Não há
Na
NDN
NÃO TENHO
.
Sugiro a inclusão, talvez no último item, sobre as puérperas em situação de vulnerabilidade
Deixar claro no 4 que não segue nenhuma orientação da equipe mesmo após diversas veze
Nada a acrescentar
Pequenas sugestões quanto à descrição: 1-Gestante ou puérpera possui suporte familiar, c

m situações de irritabilidade

que são avaliações diferentes. Pensando na graduação da avaliação, no item 2 seria: "Orientada, por

ixosa, e/ou com retraimento social. 3-Confusa ou sonolenta e/ou em crise de ansiedade, e/ou poliquei

neste número 2 e manter o 3 como uso contínuo de oxigênio por cateter ou máscara

entilação ou não, o que acho mais complexo quando estão.

esquisadoras optarem por manter, sugiro inverter o texto: Suporte respiratório: Necessidade da puérpera ou gestante de suplementação da oxigenaçã

igenação. 1-Em ar ambiente, sem necessidade de auxílio para desobstrução de vias aéreas. 2-Em ar ambiente, com necessidade de auxílio/orientaç

S CLINICOS ALTERADOS

em. No entanto foram colocados muitas situações que podem causar dúvidas no preenchimento. Sugir
sinais vitais, glicemia capilar, saturação e balanço hídrico, os outros são difíceis de mensurar em conju

sinais vitais, saturação de oxigênio, glicemia capilar, edema, balanço hídrico, avaliação de moviment

anho, como por exemplo podemos ver a paciente no puerpério imediato, ela normalmente realiza as atividades in

) descrito a seguir?

ongada.

) as pacientes auxiliadas por acompanhantes necessitam de supervisão da enfermagem. Deixaria ape
ı líquidos, e/ou necessidade de suporte por via enteral ou parenteral para suprir as necessidades diária

uso de anticoagulantes, como clexane/heparinas
2

ar nas horas de enfermagem na assistência.

s/

de que as propostas das categorias 2, 3 e 4 apresentem discriminação clara o suficiente dessas dema

uidados.

nais de uma vez ao dia e no item 4 - superior a 30 minutos ou 2 ou mais vezes ao dia.

..

a de alívio

de de cuidados frequentes de ordenha de alívio pela enfermagem.

, entendo que vai além de ter ou não rede de suporte social ou familiar
es orientadas (sempre tem!)

ompreende e adere orientações da equipe de saúde para cuidados durante a internação ou em prepa

ém ansiosa ou queixosa, e/ou retraimento social"

xosa, e/ou com irritabilidade excessiva. 4-Inconsciente e/ou sedada, demonstra desesperança e/ou co

ão e/ou de auxílio para desobstrução de vias aéreas. Visto que nas resposta a suplementação do oxigênio aparece antes da desobstrução.

ção para desobstrução de vias aéreas. 3-Com necessidade de suplementação de oxigenação por cateter ou máscara. 4-Com necessidade de ventila

o desmembrar, pois verificar somente a PA de 4/4 horas é uma demanda de trabalho diferente da dem
unto com esses dados, não sei

ações fetais, perdas via vaginal, altura ou dinâmica uterina. 1-Controles em intervalos iguais ou superio

dependente, porém neste momento ela necessita de auxílio no primeiro banho, não só orientação como dito no it

nas sem auxílio.
as. 1-Autossuficiente na ingestão de líquidos e alimentos por via oral, sem auxílio da enfermagem. 2-In

andas (p.ex.: na categoria de resposta 2 e 3 a paciente pode demandar de auxílio semelhante no que s

ro para a alta. 2-Gestante ou puérpera com ou sem suporte familiar, e/ou tem dificuldades para compr

mportamento destrutivo, e/ou luto.

ção mecânica invasiva ou não invasiva, e/ou traqueostomia.

nanda de verificar a PA de 4/4 e a glicemia capilar, por exemplo. Sugiro, no mínimo, separar "controle [illegible]

ores a 6 horas. 2-Controles em intervalos médios de 4 horas. 3-Controles em intervalos médios de 2 h[illegible]

tem 2. Só que ela também não está em repouso relativo como no item 3.

gestão de líquidos e alimentos via oral com auxílio da enfermagem. 3-Ingestão de líquidos e nutrientes

se refere aos cuidados de enfermagem, tanto auxílio para uso do banheiro quanto para uso do disposi

eender e/ou aderir às orientações da equipe de saúde para cuidados durante a internação ou em prep

de dados como sinais vitais, saturação de Oxigênio, glicemia capilar" / edema, balanço hídrico e/ou vol

oras. 4-Monitorização contínua e/ou controles em intervalos inferiores a 2h.

; por sondas, e/ou náusea/ vômitos; 4-Necessidade de nutrição e hidratação parenteral.

tivo de comadre. Na categoria de resposta 4 possivelmente a mesma estará com controle do débito ur

aro para a alta. 3-Gestante ou puérpera tem dificuldades para aderir às orientações e/ou com rede de

ume urinário / avaliação de movimentações fetais, perdas via vaginal, altura ou dinâmica uterina.

inário, o que pode ocasionar certa confusão da clareza quanto à avaliação pela(o) enfermeira(o)). Até

suporte social ou familiar que dificulta aderir às orientações da equipe de saúde durante a internação

o momento não possuo sugestão de resolução da possível falta de clareza que poderá haver nas cate

ou em preparo para a alta. 4-Gestante ou puérpera com rede de suporte social ou familiar ausente ou

egorias de resposta, fico à disposição para discussão se considerarem relevante a necessidade de rea

que a impeça de aderir às orientações da equipe de saúde durante a internação ou em preparo para a

valiar. VII- Eliminações: Necessidade de auxílio/cuidados para realizar eliminações por via fisiológica o

a alta.

ou por dispositivos. 1-Autossuficiente, frequência de eliminação urinária e intestinal normal. 2-Necessita

a de auxílio para eliminações em uso d
