# Supplementary material for: Validation of an instrument for patient classification to support obstetric nursing care
Source: Rev Bras Enferm. 2024 Jul 19;77(2):e20230401. doi: 10.1590/0034-7167-2023-0401 (PMC11259436; doi:10.1590/0034-7167-2023-0401)
Supplement: Translation

# Translation: Validação de instrumento para classificação de pacientes a fim de embasar cuidados de enfermagem obstétrica

**DOI:** 10.1590/0034-7167-2023-0401pt

**Authors:** Vanessa Damasceno Laporte, Clara Fróes de Oliveira Sanfelice, Ariane Polidoro Dini

**Affiliations:** I Universidade Estadual de Campinas. Campinas, São Paulo, Brasil

**Keywords:** Estudo de Validação, Gestão de Recursos Humanos, Avaliação em Enfermagem, Enfermagem Obstétrica, Administração Hospitalar

## Abstract

**Objetivos:**

construir e validar um instrumento para classificação de gestantes e puérperas segundo a demanda de cuidado de enfermagem.

**Métodos:**

estudo metodológico realizado em três etapas: 1) construção do instrumento com base na revisão bibliográfica; 2) validação de conteúdo aplicando a técnica Delphi com 12 especialistas; e 3) Avaliação da validade de construto convergente mediante a correlação entre os escores do instrumento construído e do instrumento de Fugulin.

**Resultados:**

foi construído um instrumento com dez indicadores de cuidados específicos para gestantes e puérperas. Obteve-se o índice de validade de conteúdo superior a 0,9, sendo necessária apenas uma rodada da técnica Delphi. O coeficiente de Spearman foi de 0,64 entre os instrumentos, indicando uma correlação forte.

**Conclusões:**

o instrumento de classificação construído especificamente para gestantes e puérperas apresentou evidências de validade de conteúdo e validade de construto convergente com instrumento amplamente utilizado em território nacional.

## INTRODUÇÃO

O ciclo gravídico-puerperal traz mudanças significativas na vida pessoal e familiar das mulheres. Além de alterações físicas e psicológicas inerentes a esse período, há mudanças no âmbito familiar e social^(1)^.

Apesar de ser um ciclo fisiológico, algumas situações tornam-no mais passível de desfechos desfavoráveis como complicações decorrentes do processo gestacional^(2)^. De acordo com o Ministério da Saúde (MS)^(3)^, aproximadamente 10% das gestações no Brasil são consideradas de alto risco. Uma gestação pode ser considerada de alto risco por diversos fatores, entre eles: hipertensão arterial, diabetes, síndromes hemorrágicas, doenças infecciosas, idade avançada ou gravidez na adolescência, neoplasias, história pregressa de parto prematuro ou má formação fetal anterior ou atual e características individuais e sociodemográficas que ofereçam risco à gestante e ao feto, além de qualquer outra condição que ponha em risco a saúde materna e/ou fetal. Em alguns casos, mulheres precisam ser internadas em leitos obstétricos para o acompanhamento ou tratamento clínico. Estudos indicam que mulheres que necessitam de internação durante a gestação por complicações obstétricas têm maior probabilidade de desfechos maternos e perinatais negativos^(2)^.

O cuidado oferecido às gestantes de alto risco no período de internação tem grande impacto na sobrevivência da mulher. A falta de planejamento para a oferta desses cuidados pode retardar tanto a implantação de medidas necessárias para reduzir o risco de morte materna quanto a melhoria na qualidade do atendimento à gestante^(4-7)^.

Visando a uma atuação eficiente da equipe de assistência, contemplando a identificação dos problemas que possam resultar em maiores danos à saúde das mulheres e seus filhos, é necessária a utilização de instrumentos discriminadores no processo de recomendar, gerar e fornecer cuidados de maneira diferenciada^(5)^.

Por sua participação ativa no cuidado a tal público, a enfermagem tem papel de destaque nessa função. No Brasil, o cálculo de dimensionamento de profissionais de enfermagem é regulamentado pelo Conselho Federal de Enfermagem (Cofen), considerando a classificação de pacientes^(8)^. A utilização de instrumentos validados para a classificação de pacientes é recomendada, de forma que devem ser consideradas as especificidades de quem recebe o cuidado de enfermagem^(9-10)^.

Diante da ausência de instrumentos de classificação de pacientes específicos para gestantes e puérperas hospitalizadas em unidades de internação obstétricas, habitualmente tem sido utilizado o instrumento de Fugulin^(11)^ para essa finalidade. Em geral, esse instrumento não permite avaliar as singularidades do cuidado específico à gestação e puerpério. Ainda que exista uma ferramenta que classifique a puérpera e o bebê de alojamento conjunto segundo a demanda de enfermagem, a classificação individual da mulher em seu período gravídico-puerperal não pode ser realizada pelo instrumento de avaliação de binômios^(12)^.

Dessa forma, a construção e validação de um instrumento que permita a classificação de gestantes e puérperas segundo o demanda de cuidados de enfermagem visa preencher uma lacuna da literatura, contribuindo diretamente com a qualificação da assistência de enfermagem obstétrica, tornando-a mais adequada e segura. Além disso, colabora para a redução da mortalidade materna, que é um dos Objetivos do Desenvolvimento do Milênio^(13)^, e para as prioridades da Agenda Nacional de Prioridades de Pesquisa em Saúde^(14)^, em que o ciclo gravídico-puerperal é um dos eixos de estudo.

## OBJETIVOS

Construir e validar um instrumento de classificação de pacientes segundo a demanda de cuidado de enfermagem às gestantes e puérperas.

## MÉTODOSS

### Aspectos éticos

O estudo atendeu às recomendações da Resolução 466/12 e foi aprovado pelo Comitê de Ética e Pesquisa do hospital onde a pesquisa foi realizada. Todos os especialistas que participaram da fase de validação de conteúdo assinaram o Termo de Consentimento Livre e Esclarecido antes da participação no estudo.

### Desenho, período e local do estudo

Trata-se de um estudo metodológico, realizado em um hospital no estado de São Paulo, de complexidade terciária e referência na assistência à saúde da mulher e do recém-nascido. O estudo ocorreu em três fases, entre outubro de 2020 e agosto de 2022:

1. Construção do instrumento (de setembro a dezembro de 2021);
2. Avaliação das evidências de validade de conteúdo por meio da técnica Delphi (de janeiro a fevereiro de 2022);
3. Avaliação da validade de construto convergente mediante a correlação entre os escores do Instrumento de Classificação de Gestantes e Puérperas construído e do instrumento de Fugulin^(11)^, em amostra de uma unidade de internação obstétrica (de abril de 2022 a agosto de 2022).

### Critérios de inclusão e exclusão

Na etapa de avaliação das evidências de conteúdo por meio da técnica Delphi, foi utilizado como critério de inclusão para a escolha dos especialistas: enfermeiros que tinham experiência em validação de instrumentos, com publicação de artigos na área; ou experiência na assistência ou gestão de enfermagem em unidades de internação obstétrica em período maior ou igual a dois anos. Os critérios de exclusão foram: não manifestar interesse ou compreensão para julgar o conteúdo do instrumento; não concordar com a assinatura do Termo de Consentimento Livre Esclarecido do protocolo de pesquisa aprovado pelo CEP; e ter experiência menor que dois anos na assistência e gestão de unidades de internação obstétrica.

O procedimento de coleta de dados para o pré-teste do instrumento foi realizado pela pesquisadora do estudo, que presta assistência no local da coleta há nove anos. Cada leito de internação obstétrica foi classificado apenas uma vez com cada instrumento no mesmo dia e horário de aplicação. Nessa fase, foi considerado o seguinte critério de inclusão: leitos onde estavam internadas gestantes e puérperas que não estavam em alojamento conjunto.

### Protocolo do estudo

O processo de construção do Instrumento de Classificação de Gestantes e Puérperas (ICGP) foi baseado em: referências de enfermagem obstétrica^(1-7)^ para identificar as potenciais áreas geradoras de demanda de cuidado de enfermagem em mulheres grávidas ou puérperas; instrumentos de classificação de pacientes^(9-12,15-17)^ previamente validados, para embasar a estrutura de avaliação de fatores, ou seja, indicadores de necessidades de cuidados de enfermagem, analisados objetivamente por um escala ordinal de quatro pontos, organizada em formato crescente em função da demanda de cuidado; e legislação para o Dimensionamento de Profissionais de Enfermagem^(8)^, que fundamentou o número de categorias de cuidado do instrumento e quantidade de horas de enfermagem por dia para cada uma das categorias de cuidado.

Após a construção do instrumento, seu processo de validação foi iniciado mediante a técnica Delphi^(17-18)^, na qual enfermeiros especialistas que atendiam aos critérios de inclusão estabelecidos julgaram o instrumento por meio do preenchimento do questionário eletrônico. O índice de validade de conteúdo (IVC) estabelecido foi de 0,8, sendo previstas repetições de coleta de dados em quantas rodadas fossem necessárias até a obtenção desse IVC ideal^(17-18)^.

O questionário foi composto por uma pergunta para avaliação da dimensionalidade, concordância e clareza do instrumento; uma pergunta relacionada ao nível da demanda de cuidados de cada um dos indicadores; e um espaço aberto para sugestões. Para avaliação das evidências de validade, pelo IVC, os especialistas atribuíram a cada indicador do instrumento uma das quatro opções: “1) Não relevante ou não apresenta clareza para avaliação da demanda de cuidados de enfermagem; ou 2) Precisa de grande revisão para ser relevante ou apresentar clareza na avaliação da demanda de cuidados de enfermagem; ou 3) Precisa de pequena revisão para ser relevante ou apresentar clareza na avaliação da demanda de cuidados de enfermagem; ou 4) Relevante e representativo na avaliação da demanda de cuidados de enfermagem”.

Depois da obtenção do consenso sobre o número de indicadores do ICGP, considerou-se a pontuação total do instrumento obtida da soma da pontuação máxima de todos os indicadores e divisão da amplitude por 5, de acordo com o número de categorias de cuidado regulamentadas pelo Cofen na Resolução sobre o dimensionamento de profissionais de enfermagem, a saber: cuidados mínimos, cuidados intermediários, cuidados de alta dependência, cuidados semi-intensivos e cuidados intensivos.

Em seguida, a versão final do ICGP foi aplicada em 120 leitos da unidade de internação obstétrica de um hospital universitário no interior do estado de São Paulo. A aplicação do ICGP foi realizada pela mesma avaliadora - que tem experiência na unidade e é uma das autoras do estudo - principalmente por causa da limitação de acesso ao local de coleta de dados no período pandêmico. Destaca-se que a planilha de coleta de dados considerou a aplicação do ICGP e do instrumento de Fugulin^(11)^, já aplicado diariamente no local de estudo. Apenas foram levados em conta os leitos ocupados por gestantes ou puérperas que não estivessem em alojamento conjunto.

### Análise dos resultados e estatística

Todas as respostas obtidas na primeira fase da técnica Delphi foram extraídas da plataforma de respostas e tabuladas em planilha eletrônica para calcular o IVC, de acordo com a fórmula^(19)^: IVC = ∑ Número de respostas “3” ou “4”/ Número total de respostas.

Os dados relacionados à aplicação do ICGP nos 120 leitos foram tabulados em planilha eletrônica, e o banco de dados foi analisado por meio do software estatístico SPSS versão 23, para realização do cálculo de validade de construto convergente do instrumento.

A validade de construto representa o grau no qual os escores de um instrumento são consistentes com hipóteses baseadas na suposição de que um instrumento mede validamente o construto a ser medido^(20)^.

Para essa avaliação, foi analisado o coeficiente de correlação de Spearman^(21)^, que é uma medida não paramétrica da correlação estatística entre os escores de duas variáveis - nesse caso, o ICGP e o instrumento de Fugulin^(11)^. Valores maiores ou iguais a 0,50 são indicativos de uma forte correlação entre as variáveis analisadas.

## RESULTADOS

Após a revisão da literatura, foi elaborada uma “Versão 0” do ICGP composta por dez indicadores de cuidado: Estado mental, comportamento e/ou humor; Suporte respiratório; Intervalo de aferição de sinais; Dependência para atividades de vida diária; Suporte alimentar e hídrico; Terapêutica medicamentosa; Eliminações; Integridade cutâneo-mucosa; Cuidados com as mamas; Rede de suporte familiar e compreensão do estado de saúde. Todos os indicadores foram compostos por escala do tipo Likert de 1 a 4 pontos em relação ao nível de demanda de cuidados de enfermagem, sendo a demanda mais baixa igual a 1 e a mais alta correspondente a 4 pontos.

Dos 15 enfermeiros especialistas convidados a julgar o conteúdo do instrumento, apenas 12 aceitaram participar da primeira fase do estudo. A caracterização dos participantes está apresentada na Tabela 1. O prazo de resposta estabelecido foi de 15 dias, porém cada questionário foi respondido em até três dias.

**Tabela 1:** Caracterização dos especialistas participantes da Avaliação do Instrumento de Classificação de Gestantes e Puérperas (N = 12), Campinas, São Paulo, Brasil, 2023

Caracterização	n	%
Idade		
28-30	1	8%
31-40	4	33%
>40	7	58%
Tempo de Formação (anos)		
6-10	4	33%
11-19	3	25%
20-27	5	42%
Tempo de experiência profissional (anos)		
4-8	2	17%
9-19	5	42%
20-26	5	42%
Área de Atuação		
Assistência direta	8	67%
Gerência	3	25%
Ensino e pesquisa	1	8%
Qualificação profissional		
Apenas graduação	1	8%
Especialização	10	83%
Mestrado	4	33%
Doutorado	3	25%

Os dados obtidos das respostas dos especialistas nessa primeira fase da técnica Delphi estão apresentados na Tabela 2.

**Tabela 2:** Porcentagem de concordância a respeito dos indicadores e situações graduadas do Instrumento de Classificação de Gestantes e Puérperas (N = 12), Campinas, São Paulo, Brasil, 2023

Indicador	Indicadores de cuidado	Situações graduadas
Rede de suporte familiar	100%	100%
Estado mental comportamento e/ou humor	100%	91,7%
Dependência para atividades de vida diária	100%	100%
Cuidado com as mamas	100%	100%
Integridade cutâneo-mucosa	100%	100%
Suporte alimentar e hídrico	100%	100%
Eliminações	100%	100%
Suporte respiratório	100%	100%
Intervalo de aferição de controles	100%	100%
Terapêutica medicamentosa	100%	100%

Todos os juízes assinalaram as opções 3 ou 4 para a análise do conteúdo; dessa forma, o IVC de todos os indicadores foi igual a 1. Além dessas respostas objetivas, alguns dos especialistas apresentaram sugestões para a melhor compreensão do instrumento, as quais foram analisadas, e aquelas julgadas pertinentes foram acatadas pelas pesquisadoras.

No indicador Estado mental, comportamento e/ou humor, um especialista sugeriu a substituição do termo “poliqueixosa” por “múltiplas queixas”. Também nesse indicador foi sugerido por outro pesquisador que a opção graduada de número 2 fosse adaptada de “Orientada e/ou pouco ansiosa ou queixosa” por “Orientada, porém ansiosa ou queixosa, e/ou retraimento social”, visto que a orientação espacial da paciente e seu comportamento são avaliações relacionadas, porém independentes. O texto foi reescrito em sua versão final como: “2 - Orientada, ansiosa ou queixosa; e/ou com retraimento social”.

Já no indicador Suporte respiratório, dois juízes apontaram a necessidade de substituição da palavra “traqueostomizada” pelo termo “com traqueostomia” na quarta graduação: “4 - Com necessidade de ventilação mecânica invasiva ou não invasiva; e/ traqueostomizada”. Foi acatada a sugestão, e a versão final ficou: “4 - Com necessidade de ventilação mecânica invasiva ou não invasiva; e/ com traqueostomia”.

Para a reformulação do indicador Dependência para atividades de vida diária, nas graduações 1 e 2, foi aceita a sugestão de dois especialistas de incluir a observação de necessidade ou não de orientação e auxílio.

As alterações realizadas no indicador Eliminações relacionaram-se à grafia das palavras “WC” na graduação de número 2 e “Ostomias” na graduação 4, conforme atualização conceitual. Foram aceitas as sugestões, e a reescrita ficou da seguinte maneira: “2 - Necessita de auxílio para eliminações no banheiro e/ou frequência de eliminações urinárias e intestinais alteradas”; e “4 - Uso de fralda, cateter vesical, estomas”.

Diante da concordância superior a 90% sobre todos os indicadores e situações graduadas, bem como IVC superior a 0,9, não houve necessidade de nova rodada da técnica Delphi. Com base nesse resultado, a versão final do ICGP foi constituída por dez indicadores de demanda de enfermagem, com as respectivas definições conceituais. Para cada indicador, foram estabelecidas quatro situações graduadas de 1 a 4 pontos, de forma crescente em função da demanda de enfermagem (Quadro 1).

**Quadro 1:** Instrumento para Classificação de Gestantes e Puérperas, Campinas, São Paulo, Brasil, 2023

Rede de suporte familiar e compreensão do estado de saúde: Necessidade de orientações e apoio à gestante, puérpera ou familiar que participa do cuidado. 1 - Gestante ou puérpera possui suporte familiar; compreende e segue as orientações da equipe de saúde para cuidados durante a internação ou em preparo para a alta. 2 - Gestante ou puérpera com ou sem suporte familiar; e/ou tem dificuldades para compreender e/ou seguir orientações da equipe de saúde para cuidados durante a internação ou em preparo para a alta. 3 - Gestante ou puérpera tem dificuldades para aderir a orientações e/ou com rede de suporte social ou familiar dificultadora para seguir orientações da equipe de saúde durante a internação ou em preparo para a alta. 4 - Gestante ou puérpera com rede de suporte social ou familiar ausente ou que impeça a adesão às orientações da equipe de saúde durante a internação ou em preparo para a alta.
**Estado mental, comportamento e/ou humor:** Avaliação das condições de orientação, comunicação não verbal do grau de atividade e/ou estado emocional da gestante ou puérpera. 1 - Orientada em tempo, espaço e pessoa; e tranquila. 2 - Orientada, porém ansiosa ou queixosa; e/ou com retraimento social. 3 - Confusa ou sonolenta; e/ou em crise de ansiedade; ou com múltiplas queixas; e/ou com irritabilidade excessiva. 4 - Inconsciente e/ou sedada; demonstra desesperança; e/ou comportamento destrutivo; e/ou luto.
**Dependência para atividades de vida diária:** Necessidade de auxílio/cuidados para mover-se, deambular, higienizar-se, vestir-se. 1 - Independente nas atividades de vida diária, deambula, se banha e realiza higiene oral sem necessidade de orientação ou auxílio. 2 - Independente nas atividades de vida diária, deambula, se banha e realiza higiene, no entanto carece de orientação e auxílio para a realização. 3 - Necessita de repouso relativo e/ou depende de cadeira de rodas e/ou órteses para locomoção segura e necessita de auxílio profissional para banhar-se e realizar higiene oral. 4 - Completamente dependente da enfermagem para as atividades de vida diária por estar acamada/contida/em repouso absoluto; realização de banho e higiene oral no leito.
**Cuidado com as mamas:** Necessidade de cuidados com as mamas em preparo para a amamentação, supressão da lactação e/ou complicações relacionadas a infecção ou patologias. 1 - Mamas flácidas sem presença de hiperemia ou com inibição eficaz da lactação quando indicado. 2 - Mamas cheias com colostro/leite à expressão sem turgidez ou dor. 3 - Mamas com sinais de ingurgitamento mamário, sem sinais de infecção, com necessidade de ordenha realizada pela puérpera. 4 - Mamas com sinais de infecção ou reação hiperálgica ou fissura mamária, com necessidade de cuidados frequentes de ordenha de alívio pela enfermagem.
**Integridade cutâneo-mucosa:** Necessidade de cuidados para manutenção ou reparação da integridade da pele ou mucosas. 1 - Pele e mucosas íntegras em toda a superfície corpórea, sem necessidades de cuidados de enfermagem para reparação. 2 - Pele desidratada ou com presença de hiperemia ou ferida operatória limpa sem sinais flogísticos, com necessidade de cuidados simples e pontuais para lubrificação da pele ou curativo de pequeno porte. 3 - Ferida operatória e/ou lesão com necessidade de curativo com repetidos cuidados de renovação no decorrer do dia e/ou tempo de realização de cuidado com a pele e mucosas superior a 15 minutos. 4 - Ferida operatória e/ou lesão com necessidade de curativo complexos com tempo de realização de cuidado com a pele e mucosas superior a 30 minutos.
**Suporte alimentar e hídrico:** Necessidade de auxílio/cuidados para ingerir alimentos e/ou líquidos e/ou necessitar de suporte por via enteral ou parenteral para suprir necessidades diárias. 1 - Autossuficiente na ingestão de líquidos e alimentos por via oral, sem auxílio. 2 - Ingestão de líquidos e alimentos via oral com auxílio. 3 - Ingestão de líquidos e nutrientes por sondas; e/ou náusea/vômitos; 4 - Necessidade de nutrição e hidratação parenteral.
**Eliminações:** Necessidade de auxílio/cuidados para realizar eliminações por via fisiológica ou por dispositivos. 1 - Autossuficiente, frequência de eliminação urinária e intestinal normal, sem necessidade de auxílio. 2 - Necessita de auxílio para eliminações no banheiro e/ou frequência de eliminações urinárias e intestinais alteradas. 3 - Uso de comadre para realizar eliminações e/ou realização de balanço hídrico e/ou controle de débito urinário. 4 - Uso de fralda, cateter vesical, estomas.
**Suporte respiratório:** Necessidade de a puérpera ou gestante precisar de auxílio para desobstrução de vias aéreas e/ou suplementação da oxigenação. 1 - Em ar ambiente, sem necessidade de auxílio para desobstrução de vias aéreas. 2 - Em ar ambiente, com necessidade de auxílio/orientação para desobstrução de vias aéreas. 3 - Com necessidade de suplementação de oxigenação, por cateter ou máscara. 4 - Com necessidade de ventilação mecânica invasiva ou não invasiva; e/ou com traqueostomia.
**Intervalo de aferição de controles:** Necessidade de observação e controle de dados como sinais vitais, saturação de oxigênio, glicemia capilar, edema, balanço hídrico, avaliação de movimentações fetais, perdas via vaginal, altura ou dinâmica uterina. 1 - Controles em intervalos iguais ou superiores a cada 6 horas. 2 - Controles em intervalos médios a cada 4 horas. 3 - Controles em intervalos médios a cada 2 horas. 4 - Monitorização contínua e/ou controles em intervalos inferiores a cada 2 horas.
**Terapêutica medicamentosa:** Necessidade de receber fármacos para tratamento ou controle de sintomas/patologias de base. 1 - Medicamentos via oral. 2 - Medicamentos via subcutânea, intramuscular e/ou endovenosa intermitente. 3 - Medicamentos via endovenosa contínua. 4 - Necessidade de fármacos vasoativos e/ou sedação e/ou hemoderivados.
**Classificação da gestante ou da puérpera** Após avaliar a mulher em todos os indicadores de cuidado, comparar com a escala de classificação para identificação da categoria de cuidado: Entre 10 e 15 pontos: cuidados mínimos Entre 16 e 21 pontos: cuidados intermediários Entre 22 e 27 pontos: cuidados de alta dependência Entre 28 e 33 pontos: cuidados semi-intensivos Maior que 40 pontos: cuidados intensivos

Após a aplicação do ICGP e do instrumento de Fugulin nos leitos de internação obstétrica, 90% dos leitos foram classificados pelo ICGP como pacientes que necessitavam de cuidados mínimos de enfermagem. Já segundo o instrumento de Fugulin, 97,5% do total de leitos avaliados representaram essa categoria de cuidado de enfermagem. A porcentagem restante de leitos foi classificada por ambos os instrumentos como pacientes que necessitavam de cuidados intermediários: 10% do total pelo ICGP e 2,5% pelo instrumento de Fugulin. Nenhum leito foi classificado com as demais categorias de cuidado na amostra avaliada.

No que se referiu à análise da validade de construto convergente do ICGP, diante da análise da equivalência entre os instrumentos de Fugulin e ICGP, obteve-se resultado do coeficiente de Spearman igual a 0,64. Isso indica uma relação positiva e de forte magnitude entre os escores dos instrumentos, isto é, em ambos os instrumentos, quanto maior o escore obtido, maior é o grau de complexidade relacionado à demanda de cuidados de enfermagem. Esse resultado mostra a validade de construto convergente do ICGP.

## DISCUSSÃO

A construção do instrumento para classificação de gestantes e puérperas seguiu a estrutura de instrumentos de classificação de pacientes previamente validados e legislação vigente para dimensionamento de profissionais de enfermagem^(8,11-12,16)^. Além disso, foi motivada pela necessidade da elaboração de um instrumento específico para pacientes gestantes e puérperas que classificasse adequadamente esse público, considerando a gestação e o puerpério como um processo da saúde da mulher que requer atenção específica^(3-4,13-14)^.

A construção do instrumento baseou-se na legislação do Conselho Federal de Enfermagem, a qual estabelece quantidade mínima de horas de assistência de enfermagem por dia em cinco categorias de cuidados: 4 horas para cuidados mínimos, 6 horas para cuidados intermediários, 10 horas para alta dependência de cuidados ou cuidados semi-intensivos e 18 horas para cuidados intensivos^(8)^.

A utilização do instrumento, como indicada pela legislação^(8)^, pode ser realizada diariamente, enquanto parte do processo de trabalho da enfermeira, para avaliar a demanda de cuidado de cada enfermaria, o que permite o planejamento da assistência de maneira mais assertiva, favorecendo o equilíbrio entre a oferta e a demanda de cuidados. Também serve como base para fundamentar os remanejamentos diários, além de permitir a análise da série histórica e do cálculo do dimensionamento de pessoal.

Para a validação, optou-se pela utilização da técnica Delphi por sua característica de preservar a confidencialidade da identidade dos participantes e possibilidade da contribuição de forma escrita por especialistas com saberes divergentes e complementares para a construção do instrumento. O emprego dessa técnica possibilita que o consenso seja derivado da reflexão sobre as questões propostas além da avaliação da clareza e relevância^(19,20,22-25)^.

Ainda que a literatura apresente como desvantagem da Técnica Delphi a grande quantidade de tempo entre o envio e devolução dos questionários, bem como a estatística de apenas 50% dos indicados responderem ao primeiro contato^(19-20,22-25)^, neste estudo alcançou-se uma taxa de retorno de 82% dos convidados em período inferior a 15 dias, e todos responderam a todas as perguntas propostas.

No que se refere à seleção dos especialistas (indispensável para a obtenção de maior qualidade na execução da técnica Delphi)^(19-20,22-25)^, foi fundamental a heterogeneidade de experiência e formação na composição do grupo de especialistas, o que permitiu avaliar o instrumento sob a perspectiva da assistência, da gestão e da pesquisa em enfermagem em unidades obstétricas.

Destaca-se que grupos de especialistas heterogêneos tendem a soluções mais qualificadas e aceitáveis. Além do mais, diante da divergência na literatura quanto ao grau de especialidade dos juízes^(19-20,22-25)^, foi imprescindível o estabelecimento do critério de inclusão de que os especialistas tivessem no mínimo dois anos de experiência em cuidado, gestão e/ou pesquisa na área obstétrica. Tal critério foi justificado pelo fato de a experiência dos enfermeiros permitir um olhar mais cuidadoso para o ICGP.

A quantidade de especialistas em estudos com a aplicação da técnica Delphi pode ser bem variável, porém pesquisas indicam que um número maior ou igual a dez especialistas, como utilizado no presente trabalho, é considerado ótimo para análise de consenso e relevância^(19-20,22-25)^.

O uso de questionários on-line como forma de coleta de dados possibilitou o agrupamento de opiniões de profissionais de diferentes localidades e experiências profissionais, levando a uma boa explanação de experiências, de acordo com a realidade das unidades obstétricas^(19-20,22-25)^.

Após criteriosa avaliação das respostas, foram realizadas modificações sugeridas pelo grupo de especialistas, sobretudo revisões para simplificar e conferir clareza na redação da versão inicial do instrumento sem, no entanto, modificar a estrutura proposta de cada indicador de cuidado e do instrumento como um todo.

Diante da concordância a respeito de todos indicadores e respectivas situações graduadas, bem como IVC = 1, foi assegurada a validade de conteúdo do ICGP^(23-25)^. Este, em sua versão final, constituiu-se de dez indicadores de demanda de cuidados de enfermagem, com suas respectivas definições conceituais. Para cada indicador, foram estabelecidas quatro situações graduadas de 1 a 4 pontos, de forma crescente em função da demanda de enfermagem.

Com a aplicação simultânea do ICGP e do Instrumento de Classificação de Pacientes de Fugulin, obteve-se validade de construto convergente com o coeficiente de correlação de Spearman superior a 0,5, considerado de forte correlação^(21)^. O valor obtido de 0,64 indicou que os dois instrumentos apresentam correlação quanto ao que é proposto a se medir: a classificação das pacientes. Destaca-se que a utilização do ICGP, por sua especificidade, pode qualificar a avaliação de enfermagem obstétrica com base na avaliação de indicadores específicos como “Cuidado das mamas” e “Rede de suporte familiar e compreensão do estado de saúde”. A avaliação específica desses indicadores é considerada essencial para o cuidado qualificado de enfermagem obstétrica, uma vez que mulheres grávidas e puérperas têm necessidades específicas de cuidado para o enfrentamento do período de internação.

Os indicadores de cuidado do Instrumento de Classificação de Gestantes e Puérperas referem se a situações específicas do período gravídico-puerperal que exigem assistência especializada por parte da enfermagem.

### Limitações do estudo

Uma limitação foi a obtenção de amostra homogênea de pacientes obstétricas em meio à mudança de perfil das pacientes obstétricas na maternidade de estudo. A partir de novembro de 2021, devido à reforma na unidade de internação intensiva e semi-intensiva de neonatologia, houve redução de leitos de internação obstétrica e revisão da referência de pacientes no sistema de saúde da região, com diminuição de complexidade na maternidade de coleta de dados. Além disso, por causa das condições pandêmicas, não foi possível coletar dados para a avaliação simultânea de enfermeiros distintos e analisar a confiabilidade interavaliadores do ICGP.

São necessários, então, estudos de confiabilidade deste instrumento e de aplicação em outras realidades que contemplem gestantes e puérperas de alto risco, para a avaliação de sua representatividade em todas as categorias de cuidado.

### Contribuições para a área da Enfermagem, Saúde ou Política Pública

Como contribuição principal, podemos citar o avanço nas pesquisas da temática de adequação e criação de instrumento específico para a classificação de gestantes e puérperas hospitalizadas, colaborando para um melhor dimensionamento de enfermagem mediante melhor categorização das necessidades de cuidado de enfermarias de internação obstétrica.

A especificidade dos indicadores relacionados ao ciclo gravídico-puerperal poderá qualificar a avaliação de enfermagem em função da demanda de cuidados de gestantes e puérperas na prática gerencial da classificação de pacientes.

## CONCLUSÕES

Este estudo permitiu a construção e disponibilização de um instrumento específico para a classificação de pacientes gestantes e puérperas em unidades de internação obstétrica nas cinco categorias de cuidado consideradas na legislação nacional para a classificação de pacientes: cuidados mínimos, cuidados intermediários, cuidados de alta dependência, cuidados semi-intensivos e cuidados intensivos.

O instrumento apresentou evidências de validade de conteúdo e validade de construto convergente. A especificidade dos indicadores relacionados ao ciclo gravídico-puerperal poderá qualificar a avaliação de enfermagem em função da demanda de cuidados de gestantes e puérperas na prática diária, tanto para a gestão operacional do cuidado quanto para a gestão tática de enfermagem, no cálculo do dimensionamento de profissionais de enfermagem.

Recomenda-se a realização de estudos visando à avaliação da validade do construto e avaliação da confiabilidade interavaliadores para posterior implementação do instrumento em enfermarias de internação obstétrica.

## Data Availability

https://doi.org/10.25824/redu/LZIFAD
